# Distribution of tetraspanins in bovine ovarian tissue and fresh/vitrified oocytes

**DOI:** 10.1007/s00418-022-02155-4

**Published:** 2022-10-15

**Authors:** Jana Jankovičová, Petra Sečová, Ľubica Horovská, Lucia Olexiková, Linda Dujíčková, Alexander V. Makarevich, Katarína Michalková, Jana Antalíková

**Affiliations:** 1 Laboratory of Reproductive Physiology, Institute of Animal Biochemistry and Genetics, Centre of Biosciences, Slovak Academy of Sciences, Dúbravská cesta 9, 84005 Bratislava, Slovak Republic; 2 Research Institute for Animal Production Nitra, National Agricultural and Food Centre, Lužianky, Slovak Republic; 3 Department of Botany and Genetics, Faculty of Natural Sciences and Informatics, Constantine the Philosopher University in Nitra, Nitra, Slovak Republic

**Keywords:** Follicle, Oogenesis, Gametes, Cryopreservation, Cluster of differentiation, Integrins

## Abstract

Tetraspanin proteins are mostly known as organizers of molecular complexes on cell membranes, widely expressed on the surface of most nucleated cells. Although tetraspanins participate in many physiological processes of mammals, including reproduction, their relevance to the processes of folliculogenesis and oogenesis has not yet been fully elucidated. We bring new information regarding the distribution of tetraspanins CD9, CD81, CD151, CD82, and CD63 at different stages of follicular development in cattle. The found distribution of tetraspanin CD9, CD63, and integrin alpha V in similar areas of ovarian tissue outlined their possible cooperation. We also describe yet-unknown distribution patterns of CD151, CD82, and CD63 on immature and mature bovine oocytes. The unique localization of tetraspanins CD63 and CD82 in the *zona pellucida* of bovine oocytes suggested their involvement in transzonal projections. Furthermore, we present an unchanged distribution pattern of the studied tetraspanins in vitrified mature bovine oocytes. The immunofluorescent analysis was supplemented by in silico data addressing tetraspanins expression in the ovarian cells and oocytes across several species. The obtained results suggest that in the study of the oocyte development and potentially the fertilization process of cattle, the role of tetraspanins and integrins should also be taken into account.

**Supplementary Information:**

The online version contains supplementary material available at 10.1007/s00418-022-02155-4.

## Introduction

Tetraspanins belong to a family of proteins characterized by a specific structure, such as four transmembrane domains containing conserved polar residues; a large extracellular loop with 4, 6, 7, or 8 conserved cysteine residues; and a small extracellular loop and short cytoplasmic tails (Boucheix and Rubinstein 2001; Yang et al. 2002; Charrin et al. 2002; Berditchevski et al. 2002; Huang et al. 2005). Extracellular domains are usually post-translationally modified by glycosylation and intracellular cysteines by palmitoylation (Stipp et al. 2003). Some tetraspanins have a tyrosine-based internalization motif in the cytoplasmic tail that is involved in protein trafficking between the plasma membrane and intracellular membrane compartments (Berditchevski and Odintsova 2007). Tetraspanins are mostly known as organizers of molecular complexes, which form the tetraspanin web at specific sites of cell membranes, known as tetraspanin-enriched microdomains (Maecker et al. 1997; Lagaudrière-Gesbert et al. 1997; Seigneuret et al. 2001). The tetraspanin web was proven to be a highly dynamic complex where tetraspanins are capable of interactions with each other (Rubinstein et al. 1996; Horváth et al. 1998) and with other transmembrane and cytosolic proteins (integrins, immunoglobulin superfamily proteins, proteoglycans, complement regulatory proteins, growth factors, growth factor receptors, and signaling enzymes) (Hemler 1998, 2001, 2005; Boucheix and Rubinstein 2001; Charrin et al. 2009) and lipids (cholesterol) (Stipp et al. 2003; Charrin et al. 2003). The physical and functional link between tetraspanins and cholesterol probably regulates the mutual interactions between tetraspanins and other partners within tetraspanin-enriched microdomains (Charrin et al. 2003; Zimmerman et al. 2016). Tetraspanins participate in many fundamental processes of cells, such as adhesion (Winterwood et al. 2006), regulation of motility, morphology, fusion, and signaling. Several tetraspanins are widely used only as markers of extracellular vesicles (Escola et al. 1998; Simpson et al. 2012; Kim et al. 2013; Andreu and Yáñez-Mó 2014) and their physiological role is often overlooked. However, it was shown that tetraspanin proteins are involved in the fertilization process of mammals, and CD9 has even been found to be essential for mouse gamete fusion (Le Naour et al. 2000; Miyado et al. 2000; Kaji et al. 2000). In mammals, a family of tetraspanins includes more than 30 members. This study was focused on five of them: CD9, CD81, CD151, CD82, and CD63. In gametes, CD9, CD81, and CD151 are the most studied tetraspanins (Jankovicova et al. 2016, 2019; Jankovičová et al. 2020a), and their involvement in sperm–egg interaction is documented at least in mice and humans (reviewed in Jankovičová et al. 2020a). Tetraspanin CD82 was included in our analysis due to its declared association with CD9 and CD81 (Horváth et al. 1998), however, immunofluorescent analysis on mammalian gametes and ovarian tissue has not yet been described. CD63-positive extracellular vesicles were proposed to play a role in ovine and human fertilization, particularly in egg implantation and mother–embryo cross-talk (reviewed in Jankovičová et al. 2020b) and the association of CD63 with CD9 was suggested (Israels and McMillan-Ward 2010). The molecular pathways that participate in the processes of folliculogenesis and oogenesis are presently not fully elucidated, even though many different molecules associated with oocytes and granulosa or theca cells have already been detected (reviewed in Jones and Shikanov 2019). This is also the case of several tetraspanins described in ovarian tissue, the exact role of which is not yet known. Takao et al. (1999) observed CD9 on the cell surface of human granulosa cells and suggested that CD9 was related to their differentiation. The expression of CD81 in mouse oocytes and granulosa cells during oogenesis was reported (Tanigawa et al. 2008). The role of CD151 on human ovarian epithelial cells (Mosig et al. 2012) in the organization of other binding partners was considered. Rapp et al. (1990) reported that CD63 mRNA as one of three highly expressed transcripts is very likely translated in human granulosa cells from ovulating follicles. If we assume that determination of tetraspanin localization is the starting point for analyzing their function and since the presence of tetraspanins CD9, CD81, CD151, CD82, and CD63 in the bovine ovarian tissue has not yet been described, the objectives of our study were to address the distribution profile of tetraspanins CD9, CD81, CD151, CD82, and CD63 in *Bos taurus* ovarian follicles at individual developmental stages.

Despite the knowledge that integrins are involved in essential reproductive processes, including gamete development (Cheng and Mruk 2002; Antosik et al. 2016), fertilization (Campbell et al. 2000; Antosik et al. 2016; Frolikova et al. 2019; Barraud-Lange et al. 2020), implantation, and placentation of many species (reviewed in Bowen and Hunt 2000; Campbell et al. 2000; Cheng and Mruk 2002; Antosik et al. 2016; Frolikova et al. 2019; Barraud-Lange et al. 2020), little is known about the regulation and function of integrin subunits in the mammalian ovary. CD9, CD81, and CD151 are tetraspanins capable of binding to the classical RGD-binding site of the integrin αVβ3 (Yu et al. 2017). Therefore, to determine the integrin alpha V relevance to ovarian physiology, we examined its localization in cow ovary together with its potential partner tetraspanin CD9. In our previous papers, we described the distribution of CD9 and CD81 in immature and mature oocytes in cattle and pigs (Jankovicova et al. 2016, 2019). Furthermore, we revealed the filament-like pattern of CD9 in the *zona pellucida* (ZP) of bovine oocytes, suggesting the involvement of this tetraspanin in transzonal projections (TZPs) (Jankovicova et al. 2019). By this study, we supplemented the data regarding localization of the tetraspanins CD151, CD63, and CD82 on oocytes isolated from antral follicles (immature) and the oocytes in vitro matured to metaphase II.

An efficient cryopreservation method of cow oocytes is essential for the protection of endangered breeds, long-term storage, and production of bovine embryos in vitro to improve the utilization of animal reproduction potential. In addition, there is an increasing interest in the human reproductive field for bovine in vitro model due to several physiological similarities between cattle and humans compared to mice (the duration of folliculogenesis and single ovulation, oocyte lipid content, embryo metabolism (Langbeen et al. 2015). It also has the advantage of an unlimited source of research material without ethical or moral restrictions. Although vitrification is a widely used technique for cow oocyte cryopreservation, it is still challenging to optimize the methodology because of many factors that affect vitrification success and post-thaw development of oocytes, resulting in low embryo cleavage rate and blastocyst development (Dujíčková et al. 2021). The oocyte cryopreservation can result in nuclear damage, including spindle disorganization and/or other ultrastructural alterations (Diez et al. 2005), whereby a major site of damage is the plasma membrane (Buschiazzo et al. 2017). Several studies have reported alterations in protein expression, localization (Wen et al. 2007; Zhou et al. 2013), or secondary structure in vitrified oocytes (Rusciano et al. 2017). However, vitrification of oocytes can also lead to changes in lipid profile (Jung et al. 2014; Leao et al. 2014) and organization (Buschiazzo et al. 2017). It was proposed that tetraspanin complexes may associate with lipid rafts, organizing signal molecules in close proximity and facilitating signal transduction (Simons and Toomre 2000; Charrin et al. 2003; Comiskey and Warner 2007; Israels and McMillan-Ward 2007; Xu et al. 2009; Huang et al. 2020). Lipid rafts and their associated proteins are proposed to play a crucial role in preimplantation developmental events (Comiskey and Warner 2007). Assuming that the organization of membrane proteins is critical for cell communication, membrane trafficking, and signaling, we examined the localization pattern of tetraspanins CD9, CD81, CD151, CD63, and CD82 in metaphase II vitrified oocytes. To provide more comprehensive information, we performed in silico analysis of tetraspanins expression in ovarian cells and oocytes using available transcriptomic and proteomic data.

## Materials and methods

All chemical reagents were obtained from Sigma-Aldrich (St. Louis, MO, USA) unless otherwise noted.

### Primary antibodies

Anti-CD63 antibody, clone CC25 (Bio-Rad, Hercules, CA, USA), mouse monoclonal IgG,1 mg/ml, working dilutions: oocytes 1:50, tissue cryosections 1:100, Western blot 1:250. Anti-CD82 antibody: ab66400 (Abcam, Cambridge, UK), rabbit polyclonal IgG, 0.6–0.9 mg/ml, working dilutions: oocytes 1:50, tissue cryosections 1:100, Western blot 1:500. Anti-CD151 antibody: ab125363 (Abcam), rabbit polyclonal IgG, 0.5 mg/ml, working dilutions: oocytes 1:25, tissue cryosections 1:50, Western blot 1:500. Anti-CD9 antibody: ABIN741015 (antibodies-online, Aachen, Germany), rabbit polyclonal IgG, 1 mg/ml, working dilutions: oocytes 1:50, tissue cryosections 1:100, Western blot 1:500. The antibody is referred to herein as pCD9. Anti-CD9 antibody: IVA50 (EXBIO Praha, a.s., Vestec, Czech Republic), mouse monoclonal IgG, 1 mg/ml, working dilution: oocytes 1:50, tissue cryosections 1:100, Western blot 1:500. This antibody is referred to herein as mCD9. Anti-CD81 (H121): sc-9158 (Santa Cruz Biotechnology, Santa Cruz, CA, USA), rabbit polyclonal IgG, 200 µg/ml, working dilutions: oocytes 1:10, tissue cryosections 1:20, Western blot 1:250. Anti-αV integrin antibody: AB1930 (Millipore, Temecula, CA, USA), rabbit polyclonal IgG, working dilutions: tissue cryosections 1:100. Anti-αVβ3 integrin antibody: MAB1976 (Millipore, Temecula, CA, USA), mouse monoclonal IgG1, 1 mg/ml. Anti-actin antibody: AB3280 (Abcam), mouse monoclonal IgG1, 0.2 mg/ml, working dilution: Western blot 1:200. The rabbit IgG isotype control (NB810-56,910) (Novus Biological, Centennial, CO, USA), 5 mg/ml working dilution: oocytes 1:250, tissue cryosections 1:500. The mouse IgG1 isotype control (clone PVV-06) (EXBIO, Vestec, Czech Republic), 1 mg/ml, working dilution: oocytes 1:50, tissue cryosections 1:100, and the mouse IgG2 isotype control (clone PVV-04) (EXBIO, Vestec, Czech Republic), 1 mg/ml, working dilution: oocytes 1:50, tissue cryosections 1:100.

Antibodies anti-CD9 (pCD9), anti-CD81, anti-CD151, and anti-CD82 were chosen based on the alignment of immunogen and target antigen sequences through the NCBI-BLAST website (Table 1).

**Table 1:** Alignment of amino acid sequences of immunogens which were used for antibody production with the corresponding sequence of the bovine molecule of tetraspanins CD9, CD81, CD151, and CD82

Tetraspanin	CD9	CD81	CD151	CD82			
Antibody	ABIN741015 (antibodies-online, Aachen, Germany)	sc-9158 (Santa Cruz Biotechnology, Santa Cruz, CA, USA)	ab125363 (Abcam, Cambridge, UK)	ab66400 (Abcam, Cambridge, UK)			
Immunogen	Synthetic peptide corresponding to 120–165 amino acids of human CD9	Peptide corresponding to 90–210 amino acids of human CD81	Synthetic peptide corresponding to 154–203 amino acids of human CD151	Synthetic peptide corresponding to amino acids 250 to the C-terminus of human CD82			
UniProt identifier	P30932	Q3ZCD0	Q3ZBH3	A0A3Q1NA52 _BOVIN	A5D7E6 _BOVIN	A0A3Q1NBQ9 _BOVIN	F6QCC9 _BOVIN
Identity (%)	79	91	86	94	80	94	80
Positivity (%)	90	96	92	93	80	93	80
Molecular weight (kDa)	25.258	25.854	27.987	36.550	30.019	29.863	40.529

Similarities between amino acid sequences of immunogens which were used for antibody production and the corresponding sequence of a bovine molecule of tetraspanins CD9, CD81, CD151, and CD82 were detected using The Basic Local Alignment Search Tool (BLAST®) (https://blast.ncbi.nlm.nih.gov/Blast.cgi). UniProt identifier of protein https://www.uniprot.org/) (UniProt Consortium 2021). Monoclonal antibody anti-CD9 antibody: IVA50 (EXBIO Praha, a.s., Vestec, the Czech Republic) This antibody is referred to herein as mCD9. Anti-CD63 antibody, clone CC25 (Bio-Rad, Hercules, CA, USA) was produced directly to bovine CD63 molecule

### Secondary antibodies

Goat anti-mouse IgG (H + L) secondary antibody, Alexa Fluor 488, preadsorbed: ab150117 (Abcam), 2 mg/ml, working dilution 1:500. Donkey anti-rabbit IgG (H + L) secondary antibody, Alexa Fluor 488, preadsorbed: ab150061 (Abcam), 2 mg/ml, working dilution 1:500. Donkey anti-rabbit IgG (H + L) highly cross-adsorbed secondary antibody, Alexa Fluor 647: A-31571 (Thermo Scientific, Rockford, IL, USA), 2 mg/ml, working dilution 1:500. Horse anti-mouse IgG conjugated to horseradish peroxidase (HRP) (Vector Laboratories, Burlingame, CA, USA) working dilution 1:5000, and anti-rabbit IgG conjugated to horseradish peroxidase (HRP) (Millipore, Temecula, CA, USA), working dilution 1:10,000.

### Ovarian tissue processing and analysis

#### Western-blot analysis

Samples of ovarian cortical tissue obtained from undefined cows at a local slaughterhouse (Malá Mača, Slovak Republic) were homogenized in 1% (v/v) Triton X-100 in Tris–HCl buffer (pH 6.8) with 0.5% Protease Inhibitor Cocktail by homogenizer (UltraTurrax T18 IKA-Labortechnik, Germany). The homogenate was left on ice for 1 h to extract the proteins and subsequently centrifuged at 10,000 × *g* for 10 min at 4 °C. The protein extract was separated by 12% SDS-PAGE under non-reducing (detection of CD9 and CD63) or reducing (detection of CD81, CD151, and CD82) conditions and transferred onto nitrocellulose membranes (Advantec Toyo Kaisha Ltd., Tokyo, Japan). The molecular weights of the separated proteins were estimated using PageRuler Plus Prestained Protein Ladder (Thermo Scientific, Rockford, IL, USA). After blocking with 5% non-fat milk (SERVA Electrophoresis GmbH, Heidelberg, Germany) in 0.1% Tween 20 in PBS, the membranes were incubated with primary antibodies: monoclonal (anti-CD9 and anti-CD63) and polyclonal (anti-CD9, anti-CD81, anti-CD151, and anti-CD82) overnight at 4 °C, followed by 1 h of incubation with anti-rabbit/mouse IgG-HRP conjugate. The antibody reaction was visualized using SuperSignal West Pico Chemiluminescent Substrate (Thermo Scientific, Rockford, IL, USA). The HRP chemiluminescence was monitored with VWR^®^ Imager CHEMI Premium detection systems and analyzed using the VWR^®^ Image Capture Software (VWR International, Radnor, PA, USA).

### The immunofluorescent assay

The ovaries were isolated from undefined cows at a local slaughterhouse (Malá Mača, Slovak Republic). Ovarian tissue stored in TissueTek (Sakura Finetek, Alphen aan den Rijn, NL) at − 80 °C was used to prepare cryosections (5 µm) on a Leica Cryocut 1800 cryostat (Leica Microsystems, Wetzlar, Germany) that were subsequently fixed for 5 min in wet cold acetone-methanol (1:1) solution and dried. All treatments were performed in a humid chamber to prevent the cell smears from drying out. Samples were blocked with Super Block Blocking Buffer (Thermo Scientific, Rockford, IL, USA) for 1 h at 37 °C and treated with the primary antibody for 1 h at 37 °C. A secondary antibody diluted in saline was applied for 30 min in the dark at room temperature. Nuclear DNA was stained with Vectashield mounting medium containing DAPI (Vector Laboratories, Burlingame, CA, USA). Immunostaining was evaluated under a Leica DM5500 B epifluorescence microscope at 100× and 400× magnifications, and fluorescence images were acquired using a Leica DFC340 FX digital camera and processed using Leica Advanced Fluorescence software. The settings for the epifluorescence microscope were fixed for every image. None of the images have been manipulated.

Follicles were classified based on their morphology and diameter size as follows: primordial follicles: < 39 µm; primary follicles: 40–55 µm; secondary follicles: 56–250 µm; and tertiary follicles: > 250 µm (Rosales-Torres et al. 2012; Paulini et al. 2014).

### Co-immunoprecipitation analysis

Sections of fresh-frozen ovarian cortical tissue obtained from undefined cows at a local slaughterhouse (Malá Mača, Slovak Republic) were homogenized in 1% (v/v) Triton X-100 in PBS with 0.5% Protease Inhibitor Cocktail by homogenizer (UltraTurrax T18 IKA-Labortechnik, Germany). The homogenate was left on ice for 1 h to extract the proteins and subsequently centrifuged at 10,000 × *g* for 10 min at 4 °C. Then the supernatant was incubated with anti-CD9, anti-CD63, and anti-αVβ3 monoclonal antibodies, in the final amount of 1 µg per sample for 4 h at RT in the rotator. As a control, rabbit IgG1 in the same concentration was used. Then, 20 µl of Protein G Agarose (Thermo Scientific) was added and incubated overnight at 4 °C in the rotator. Precipitates bound to Protein G were washed 5 × in PBS with 0.5% Protease Inhibitor Cocktail for 5 min at 4 °C in the rotator. Co-immunoprecipitated complexes were eluted from Protein G Agarose by incubation in the non-reducing sample buffer for 5 min at 100 °C. After the electrophoretic separation in 12% polyacrylamide gel and transfer onto nitrocellulose membrane (Advantec Toyo Kaisha Ltd.), the membrane was blocked with 5% non-fat milk (SERVA) in 0.1% Tween 20 in PBS. The membranes with CD9, and CD63 immunoprecipitates were incubated with anti-αV integrin antibody; αVβ3 immunoprecipitate with anti-CD63 antibody, overnight at 4 °C, followed by 1 h of incubation with anti-rabbit/mouse IgG-HRP conjugate. The antibody reaction was visualized using SuperSignal West Pico Chemiluminescent Substrate (Thermo Scientific). The HRP chemiluminescence was monitored with VWR^®^ Imager CHEMI Premium (VWR International) detection systems and analyzed using the VWR^®^ Image Capture Software.

### Oocyte processing and analysis

#### Isolation and maturation of oocytes

Cumulus-oocyte complexes (COCs) were aspirated from the antral follicles (2–8 mm) of the ovaries from slaughtered cows transported to the laboratory in physiological solution at 37 °C. COCs with a compact cumulus and homogenous ooplasm referred to as immature oocytes were selected under an Olympus SD30 stereomicroscope. Maturation of oocytes to metaphase II was performed according to Makarevich and Markkula (2002). Cumulus-oocyte complexes were matured in 500 µl of TCM-199 medium with GlutaMAX™ (Invitrogen, Darmstadt, Germany) with 10% fetal calf serum (FCS), 0.025 mol/l sodium pyruvate (27.5 mg/ml saline), Pluset (mixture of FSH and LH 25 IU/ml; Minitube, Čeladice, Slovak Republic) and gentamicin (40 mg/ml) for 21 h at 39 °C in a humidified atmosphere with 5% CO_2_ without mineral oil cover. The oocytes were then analyzed by immunofluorescent assay and Western blot.

### Vitrification of oocytes

COCs intended for vitrification were matured for 21 h and those selected for the control group were matured for 23 h in a maturation medium at 38.5 °C and 5% CO_2_.

For cryopreservation of in vitro matured oocytes, an ultra-rapid cooling technique in a minimum volume was used (Olexiková et al. 2019). Selected matured oocytes were stripped of excessive cumulus layers by vortexing for 30 s. Oocytes with approximately three remaining cumulus cell layers were placed into equilibration solution (3% ethylene glycol (EG) in M199-HEPES medium supplemented with 10% FCS) for 12 min. After equilibration, the oocytes were transferred to vitrification solution (30% EG + 1 mol/l sucrose in M199-HEPES medium with 10% FCS) at room temperature for 25 s. The oocytes (10–15) in a small drop were placed with a glass micropipette onto 300-mesh nickel electron microscopy grids. Any excess medium was removed with filter paper, and then the oocytes were immediately plunged into liquid nitrogen for storage (several weeks).

For warming, nickel grids were directly transferred into a thawing solution (0.5 M sucrose in M199-HEPES medium, at 37 °C) for 1 min. The warmed oocytes were transferred across the three diluent solutions (0.25 mol/l, 0.125 mol/l and 0.0625 mol/l sucrose in M199-HEPES) for 3 min in each, and then washed twice in M199-HEPES medium with 10% FCS for 5 min. Oocyte survival was evaluated based on the integrity of the ooplasm and the *zona pellucida* after 2 h post-thawing culture. Immunofluorescence and Western-blot experiments were performed only on surviving oocytes.

### The immunofluorescent analysis

Immature oocytes were washed in PVA-Dulbecco’s PBS (1 mg/ml), and cumulus cells were removed by 5 min of vortexing in hyaluronidase type IV-S from bovine testes (150 U/ml). Cumulus cells from mature oocytes were removed by 5 min of vortexing in PVA-Dulbecco’s PBS (1 mg/ml) and in the case of vitrified oocytes for 30 s in hyaluronidase. Oocytes were fixed in 3.7% paraformaldehyde in PBS for 7 to 15 min at room temperature and incubated with the primary antibody diluted in PVA-Dulbecco’s PBS for 45 min at 39 °C. A secondary antibody was applied for 30 min at room temperature in the dark. Then the oocytes were placed on slides and mounted in Vectashield mounting medium with DAPI (Vector Laboratories). The samples were evaluated under a Leica DM5500 B epifluorescence microscope at 400 × magnification, and fluorescence images were acquired using a Leica DFC340 FX digital camera and processed using Leica Advanced Fluorescence software. The settings for the epifluorescence microscope were fixed for every image. None of the images were manipulated. Representative results are shown.

### Western-blot analysis

The fresh mature and vitrified oocytes were solubilized in reducing sample buffer, boiled for 5 min, separated in 12% SDS-PAGE (30 oocytes per lane), and transferred onto nitrocellulose membranes. After blocking with 5% non-fat milk in 0.1% Tween 20 in PBS, the membranes were incubated with primary antibodies: anti-CD9, anti-CD81, anti-CD151, anti-CD82, and anti-CD63 overnight at 4 °C, followed by 1 h of incubation with anti-rabbit/mouse IgG-HRP conjugate. Detection of actin was used as a loading control. The antibody reaction was visualized using SuperSignal West Pico Chemiluminescent Substrate. The HRP chemiluminescence was monitored with VWR^®^ Imager CHEMI Premium detection systems and analyzed using the VWR^®^ Image Capture Software.

Densitometric analysis was performed using ImageJ software. The relative density of individual tetraspanins in fresh and vitrified mature oocytes was calculated as the ratio of the optical density of antibodies to tetraspanins and actin in the blot. Results are presented as the mean ± SEM of three replicates. The statistical significance of differences was evaluated by Student’s* t* test using the statistical software Sigma Plot 11.0.

### In silico analysis of CD9, CD81, CD151, CD63, and CD82 transcriptome with expression in cells of cattle, human, pig, and mouse ovary and oocyte

In silico analysis was performed using the following data sources: genes for tetraspanins CD9, CD81, CD151, CD63, and CD82 were retrieved from the Bgee database (release 14.2) (http://bgee.org/) (Bastian et al. 2020). The appropriate transcripts and UniProt Matches were obtained from the Ensembl database (release 104-May 2021) (https://www.ensembl.org/) (Howe et al. 2021). Transcripts and genes were listed in Ensembl ID format. UniProt Match referred to the UniProt identifier of protein (https://www.uniprot.org/) (UniProt Consortium 2021) that corresponds to the Ensembl transcript. Only transcripts encoding the protein were included. Expression data (expression, rank scores, and expression scores) were retrieved from the Bgee database (release 14.2). Rank scores of expression calls reported in the database are normalized across genes, conditions, and species. A low score means that the gene is highly expressed in the condition. Max rank score in all species: 4.10e4. Min rank score varies across species. Expression scores of expression calls use the minimum and maximum rank of the species to normalize the expression to a value between 0 and 100. A low score means that the gene is lowly expressed in the condition.

## Results

### Detection of tetraspanins CD9, CD81, CD151, CD82, and CD63 in cow ovarian tissue

**Fig. 1:**
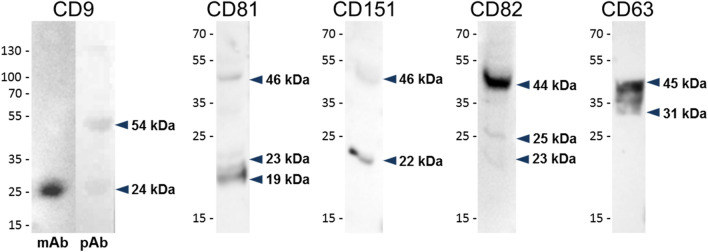
The reaction of antibodies with protein extracted from cow ovarian tissue. Tissue was analyzed by Western-blot analysis after protein separation by SDS-PAGE (12% gel). Monoclonal anti-CD9 antibody (mAb) detected a band of ~ 24 kDa and an additional ~ 54-kDa band was detected by polyclonal anti-CD9 antibody (pAb). Polyclonal anti-CD81 antibody reacted with proteins of 19, 23, and 46 kDa. Anti-CD151 polyclonal antibody recognized two bands of ~ 22 and ~ 46 kDa, anti-CD82 polyclonal antibody detected bands ~ 23, 25, and 44 kDa, and monoclonal anti-CD63 antibody revealed the broad band from ~ 31 to 45 kDa

The presence of all tetraspanins in cow ovarian tissue was confirmed by immunoblotting of protein extracts separated by SDS-PAGE (Fig. 1). Monoclonal anti-CD9 antibody recognized band of approximately 24 kDa and polyclonal anti-CD9 antibody an additional band of 54 kDa. Antibody against CD81 molecule detected strong band with a molecular mass of 19 kDa and additional weak bands of approximately 23 and 46 kDa. The immunodetection with anti-CD151 antibody revealed bands with a molecular mass of 22 and 46 kDa. Anti-CD82 antibody reacted with a band of 44 kDa and with additional weak bands of approximately 25 and 23 kDa and monoclonal anti-CD63 antibody recognized broad band from 31 to 45 kDa.

**Fig. 2:**
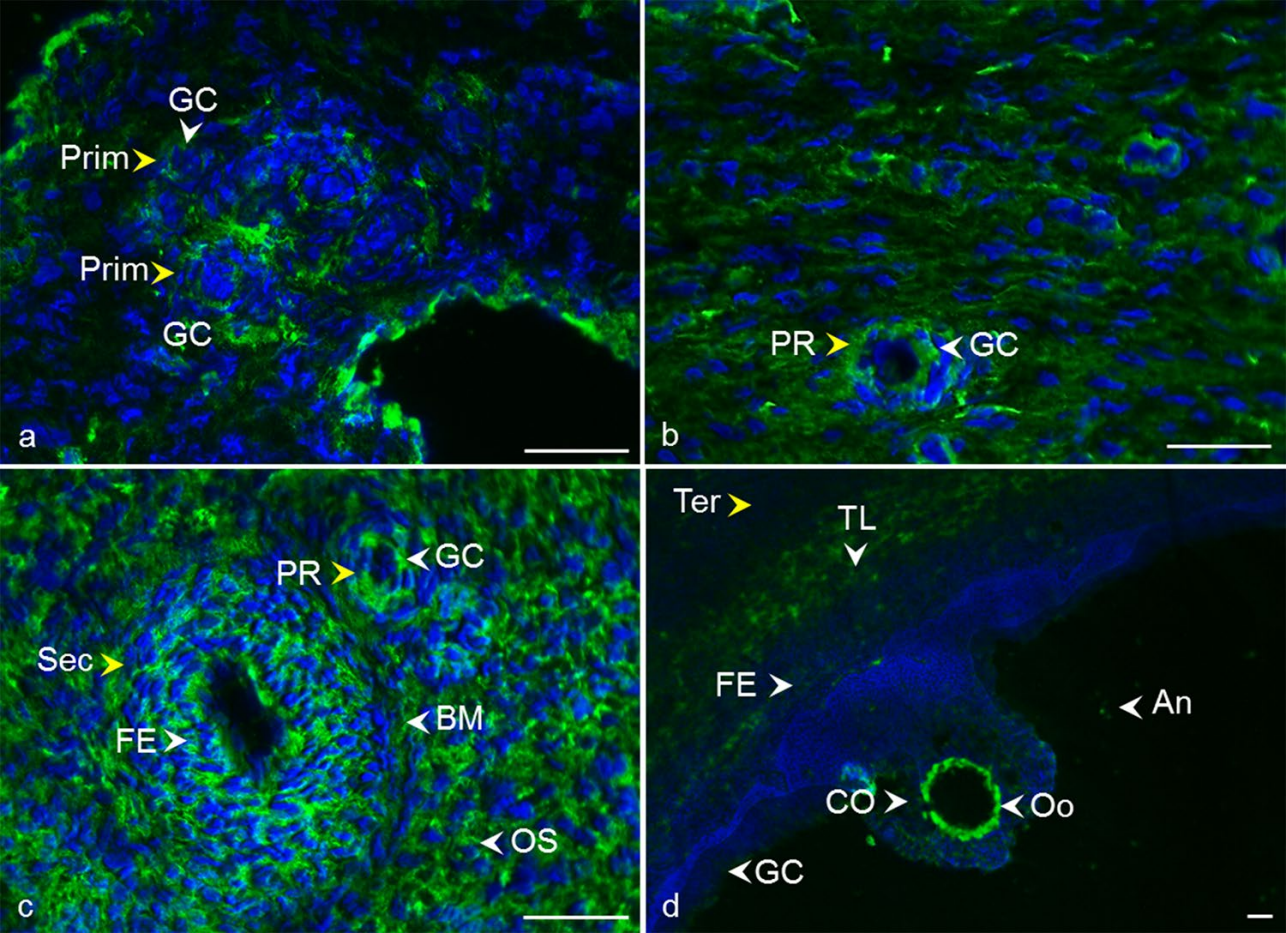
Localization of CD9 tetraspanin detected by monoclonal anti-CD9 antibody in cow ovarian tissue. Primordial follicle (Prim) (**a**) and primary follicle (PR) **b** CD9 in the area of oocyte and granulosa cells (GC). Secondary follicle (Sec) **c** CD9 in follicular epithelium (FE), basement membrane (BM), and ovarian stroma (OS). Tertiary follicle (Ter) **d** CD9 in follicular epithelium (FE), theca layers (TL), granulosa cells (GC), cumulus oophorus (CO), oocyte area (Oo), and antrum (An).* Yellow arrows* refer to the follicle stage and the* white arrows* point to the localization of tetraspanin. CD9 (*green*), DNA (*blue*). The* scale bar* represents 50 µm

**Fig. 3:**
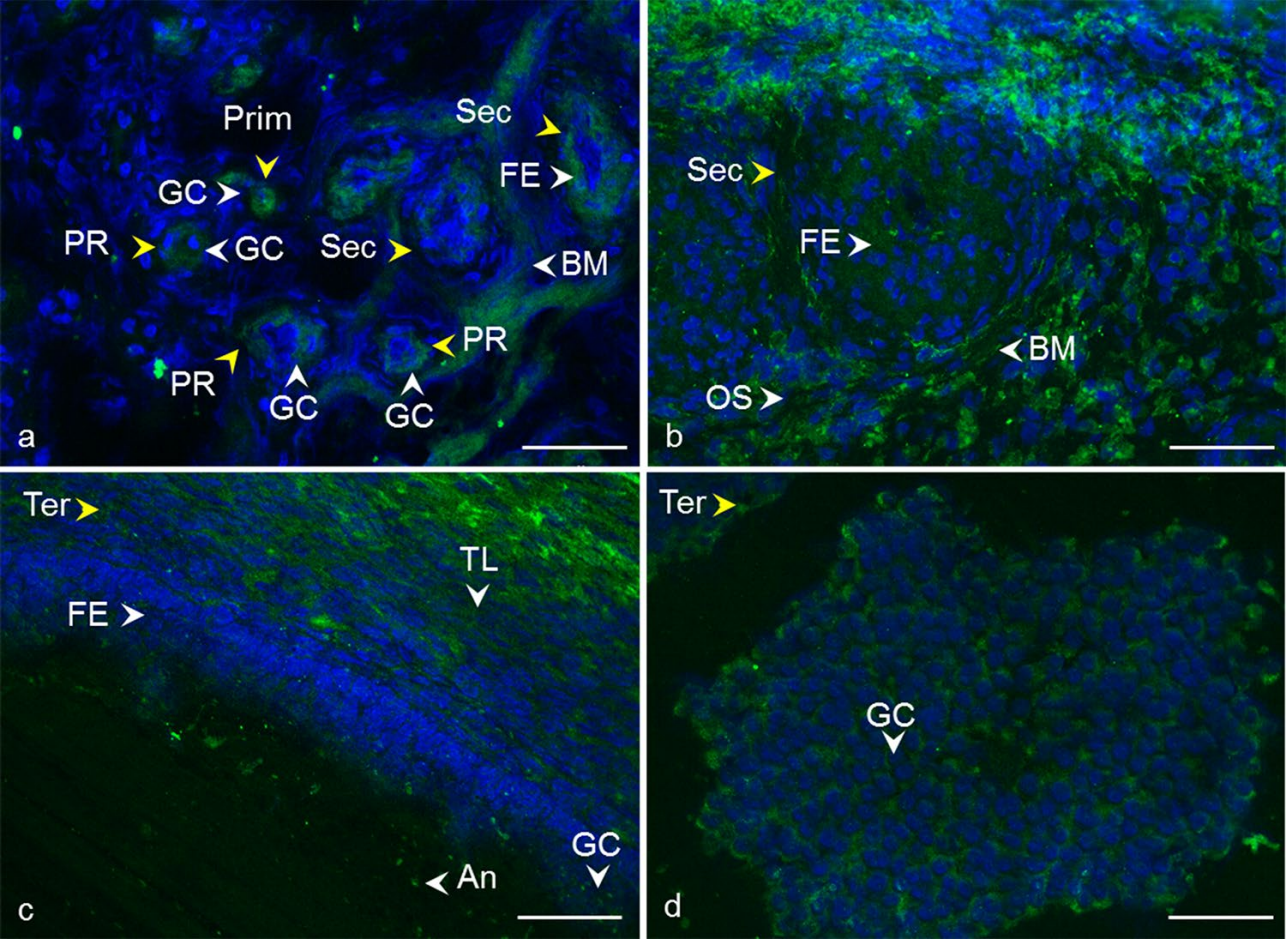
Localization of CD9 tetraspanin detected by polyclonal anti-CD9 antibody in cow ovarian tissue. Primordial follicle (Prim) and primary follicle (PR) **a** CD9 in the area of oocyte and granulosa cells (GC). Secondary follicle (Sec) **a**, **b** CD9 in follicular epithelium (FE), basement membrane (BM), and ovarian stroma (OS). Tertiary follicle (Ter) **c**, **d** CD9 in follicular epithelium (FE), theca layers (TL), granulosa cells (GC), and antrum (An).* Yellow arrows* refer to the follicle stage and the* white arrows* point to the localization of tetraspanin. CD9 (*green*), DNA (*blue*). The* scale bar* represents 50 µm

**Fig. 4:**
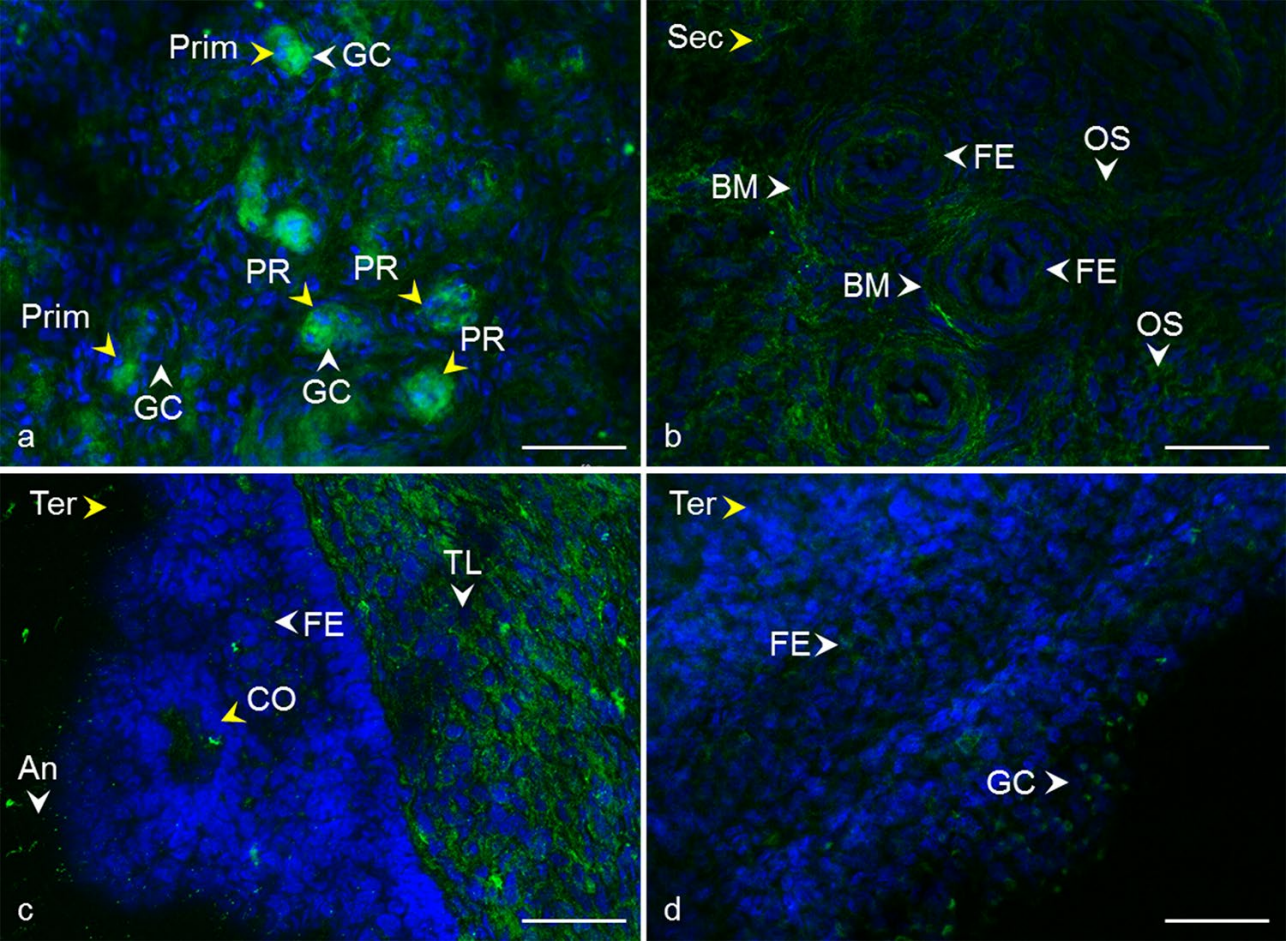
Localization of CD81 tetraspanin in cow ovarian tissue. Primordial follicle (Prim) and primary follicle (PR) **a** CD81 in the area of oocyte and granulosa cells (GC). Secondary follicle (Sec) **b** CD81 in follicular epithelium (FE), basement membrane (BM), and ovarian stroma (OS). Tertiary follicle (Ter) **c**, **d** CD81 in follicular epithelium (FE), theca layers (TL), cumulus oophorus (CO), antrum (An), and granulosa cells (GC).* Yellow arrows* refer to the follicle stage and the* white arrows* point to the localization of tetraspanin. CD81 (*green*), DNA (*blue*). The* scale bar* represents 50 µm

**Fig. 5:**
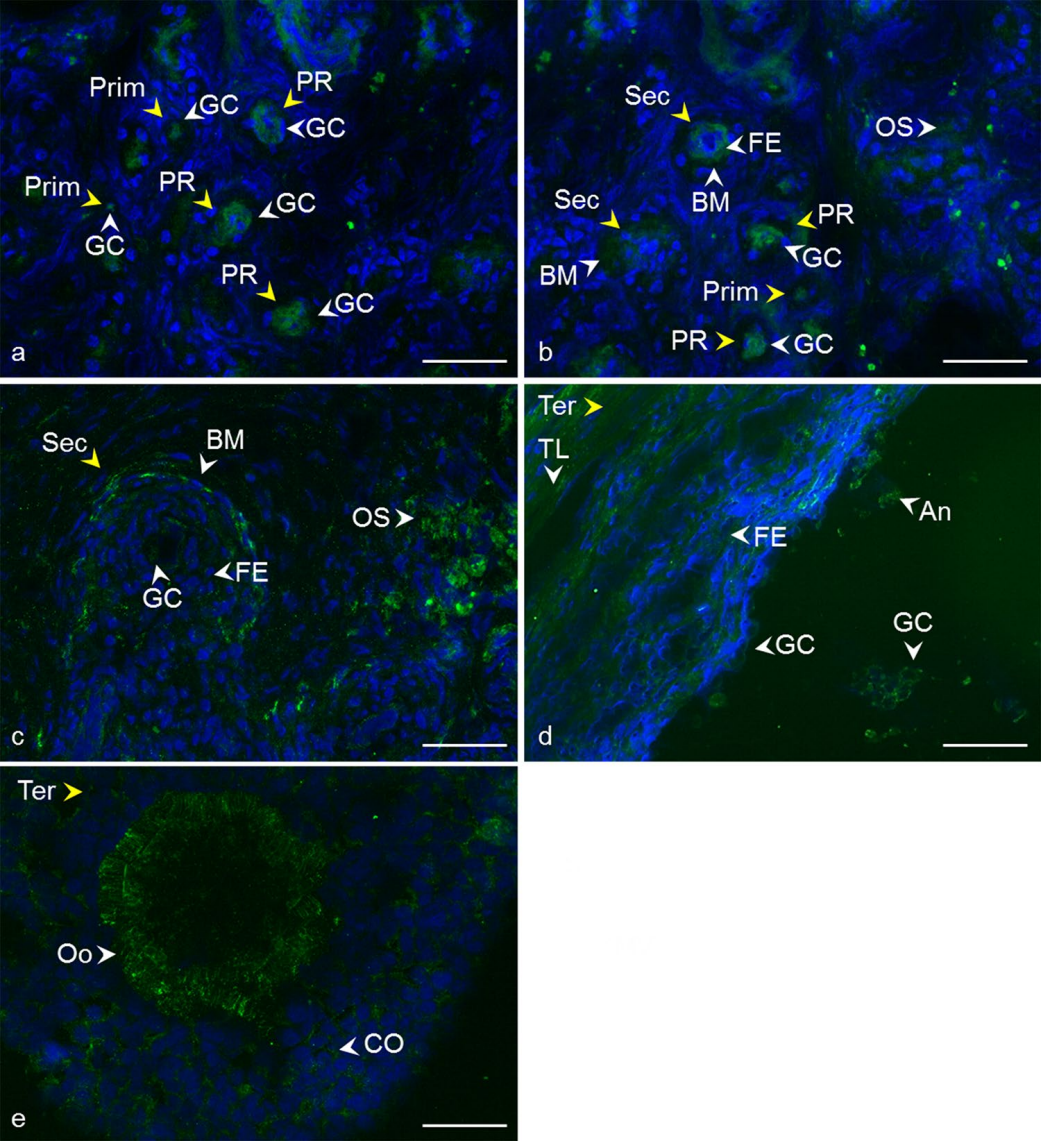
Localization of CD151 tetraspanin in cow ovarian tissue. Primordial follicle (Prim) and primary follicle (PR) **a**, **b** CD151 in the area of oocyte and granulosa cells (GC). Secondary follicle (Sec) **b**, **c** CD151 in follicular epithelium (FE), basement membrane (BM), and ovarian stroma (OS). Tertiary follicle (Ter) **d**, **e** CD151 in follicular epithelium (FE), theca layers (TL), granulosa cells (GC), cumulus oophorus (CO), oocyte area (Oo), and antrum (An).* Yellow arrows* refer to the follicle stage and the* white arrows* point to the localization of tetraspanin. CD151 (*green*), DNA (blue). The* scale bar* represents 50 µm

**Fig. 6:**
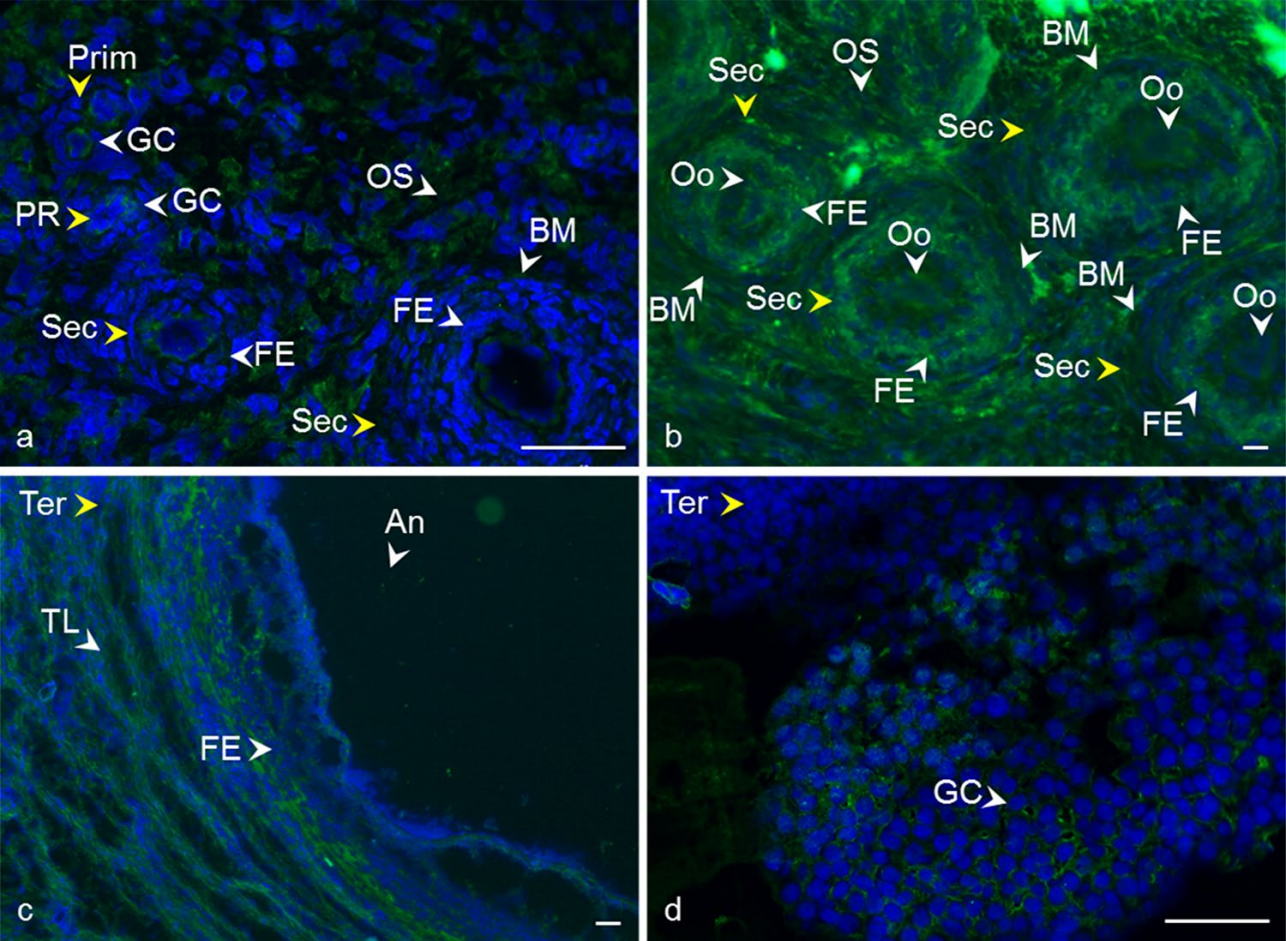
Localization of CD82 tetraspanin in cow ovarian tissue. Primordial follicle (Prim) and primary follicle (PR) **a** CD82 in the area of oocyte and granulosa cells (GC). Secondary follicle (Sec) **a**, **b** CD82 in oocyte area (Oo), the follicular epithelium (FE), basement membrane (BM), and ovarian stroma (OS). Tertiary follicle (Ter) **c**, **d** CD82 in follicular epithelium (FE), theca layers (TL), granulosa cells (GC), and antrum (An).* Yellow arrows* refer to the follicle stage and the* white arrows* point to the localization of tetraspanin. CD82 (*green*), DNA (*blue*). The* scale bar* represents 50 µm

**Fig. 7:**
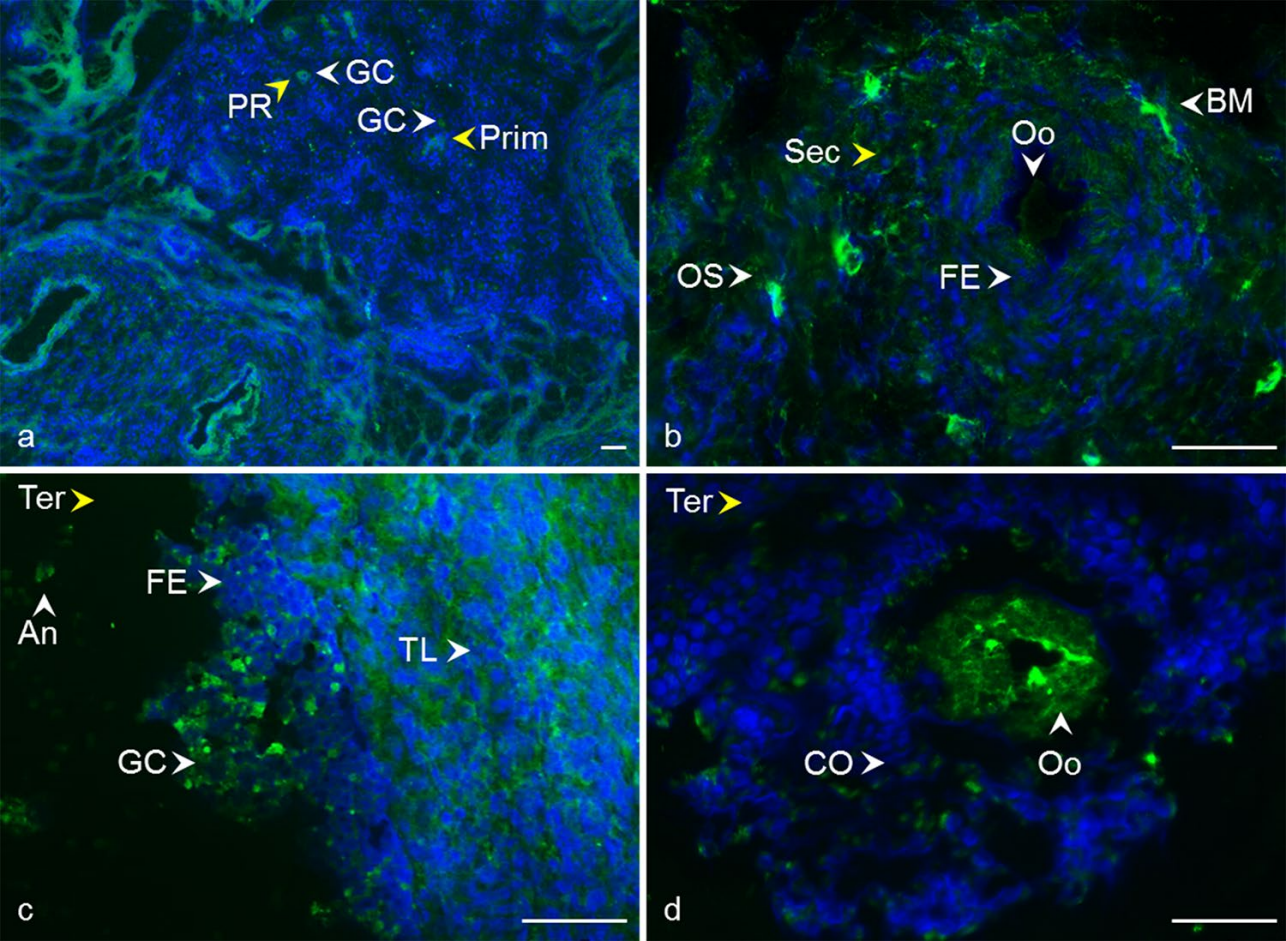
Localization of CD63 tetraspanin in cow ovarian tissue. Primordial follicle (Prim) and primary follicle (PR) **a** CD63 in the area of oocyte and granulosa cells (GC). Secondary follicle (Sec) **b** CD63 in oocyte area (Oo), the follicular epithelium (FE), basement membrane (BM), and ovarian stroma (OS). Tertiary follicle (Ter) **c**, **d** CD63 in follicular epithelium (FE), theca layers (TL), granulosa cells (GC), cumulus oophorus (CO), oocyte area (Oo), and antrum (An).* Yellow arrows* refer to the follicle stage and the* white arrows* point to the localization of tetraspanin. CD63 (*green*), DNA (*blue*). The* scale bar* represents 50 µm

After immunofluorescent analysis of ovarian tissue, a similar localization was observed for all tetraspanins (CD9, CD81, CD151, CD82, and CD63) analyzed at different follicle developmental stages (Fig. 2, 3, 4, 5, 6, 7). The results are also summarized in Table 2. In the primordial and primary follicles, immunoreactivity was concentrated mainly in the area of oocytes and pre-granulosa cells, and granulosa cells. At the secondary follicle stage, a recognizable signal was observed in the follicular epithelium and the basement membrane. The immunoreactivity was also revealed between the cells of the surrounding ovarian stroma. A clear signal in the oocyte area was observed for tetraspanins CD82 and CD63. In tertiary follicles, all tetraspanins were detected in the follicular epithelium and the surrounding theca layers. We also observed intense signal in the area of the granulosa cells and moderate labeling in the antrum. Apparent immunoreactivities for tetraspanins CD9 (detected by mCD9), CD63, and CD81 were found among the *cumulus oophorus* cells. For tetraspanins CD9 and CD63, a recognizable signal was also observed in the *zona pellucida*. Negative controls prepared for all samples showed no immunoreactivity (Supplementary Fig. 1, 2).

**Table 2:** Localization of tetraspanins CD9, CD81, CD151, CD82, and CD63 in cow ovarian tissue, follicles, and oocytes

Follicle stage	CD9	CD81	CD151	CD82	CD63
Primordial	Oocyte area, granulosa cells	Oocyte area, granulosa cells	Oocyte area, granulosa cells	Oocyte area, granulosa cells	Oocyte area, granulosa cells
Primary	Oocyte area, granulosa cells	Oocyte area, granulosa cells	Oocyte area, granulosa cells	Oocyte area, granulosa cells	Oocyte area, granulosa cells
Secondary	Follicular epithelium, basement membrane, ovarian stroma	Follicular epithelium, basement membrane	Follicular epithelium, basement membrane	Follicular epithelium, basement membrane, oocyte area	Follicular epithelium, basement membrane, oocyte area
Tertiary	Follicular epithelium, theca layers, granulosa cells, antrum, cumulus cells, oocyte area	Follicular epithelium, theca layers, granulosa cells, antrum, cumulus cells	Follicular epithelium, theca layers, granulosa cells, antrum, oocyte area	Follicular epithelium, theca layers, granulosa cells, antrum	Follicular epithelium, theca layers, granulosa cells, antrum, cumulus cells, oocyte area
Oocyte	CD9	CD81	CD151	CD82	CD63
Immature	mCD9: PM, filament-like structures passing through the ZP (Jankovicova et al. 2019), pCD9: the interrupted line on the PM with partial extension into the PVS (Jankovicova et al. 2019)	Clusters located along with the PM (Jankovicova et al. 2016)	Clusters lining up along the PM and/or in the PVS	Clusters in or near the PM filaments overlapping the ZP	Clusters in or near the PM filaments overlapping the ZP
Mature	mCD9: PM, filament-like structures passing through the ZP (Jankovicova et al. 2019), pCD9: the interrupted line on the PM with partial extension into the PVS (Jankovicova et al. 2019)	Clusters located along the PM (Jankovicova et al. 2016)	Clusters appertained to the PM	Clusters in or near the PM filaments overlapping the ZP, outer margin of the ZP	Clusters in or near the PM, filaments overlapping the ZP
Vitrified mature	mCD9: PM, filament-like structures passing through the ZP pCD9: the interrupted line on the PM with partial extension into the PVS	Clusters located along the PM	Clusters appertained to the PM	Clusters in or near the PM, filaments overlapping the ZP, outer margin of the ZP, cumulus cells	Clusters in or near the PM, filaments overlapping the ZP

*mCD9* monoclonal anti-CD9 antibody, *pCD9* polyclonal anti-CD9 antibody, *PM* plasma membrane, *ZP zona pellucida*, *PVS* perivitelline space

### Co-localization of tetraspanins CD9, CD63, and alpha V integrin in cow ovarian tissue

**Fig. 8:**
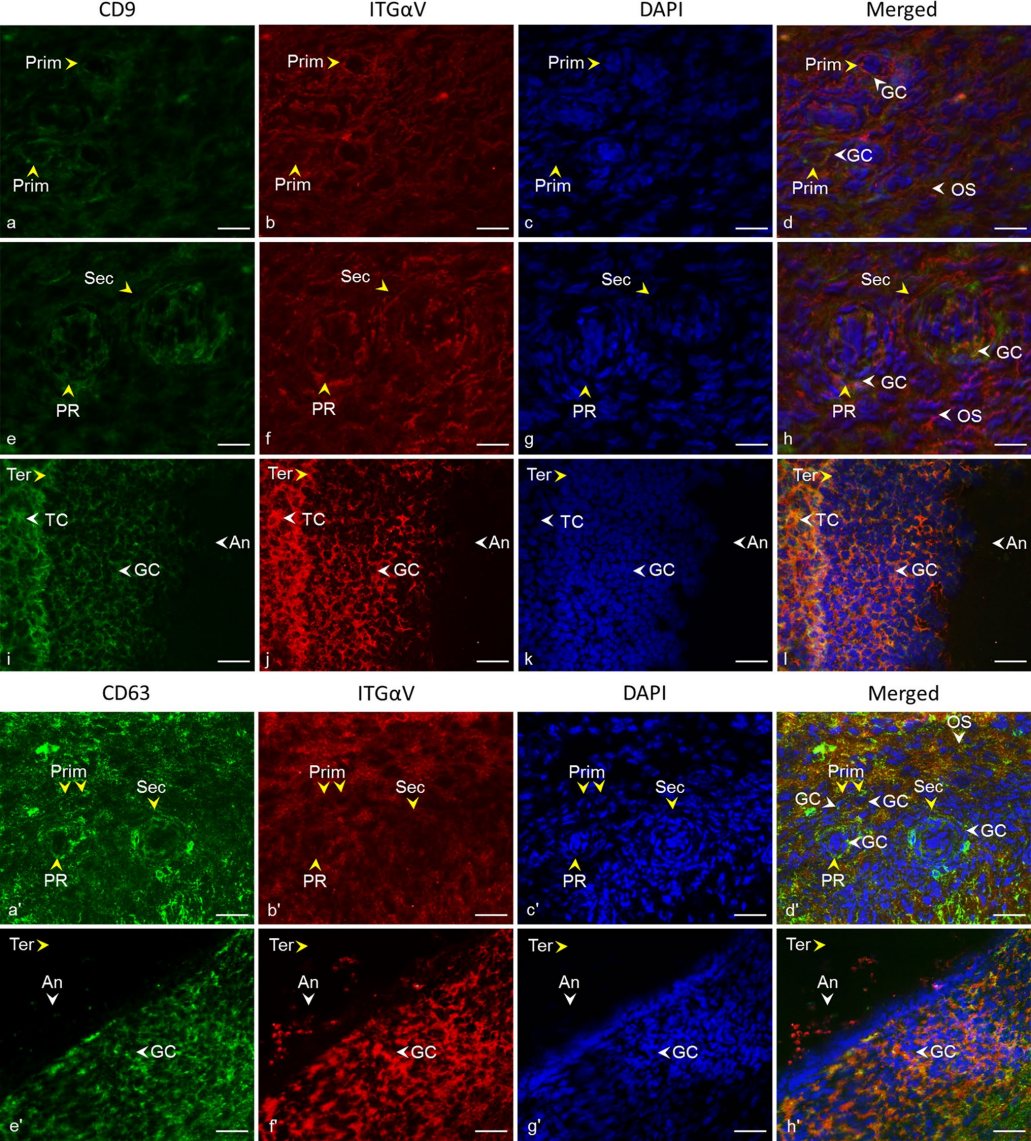
Localization of CD9 and CD63 tetraspanins and alpha V integrin in cow ovarian tissue.* White arrows* point to similar localization of CD9 tetraspanin (*green*) and alpha V integrin (*red*) in granulosa cells (GC) of primordial follicles (Prim) (**a**–**d**), the primary follicle (PR), the secondary follicle (Sec) (**e**–**h**), and surrounding ovarian stroma (OS) (**d**, **h**), and in theca cells (TC), granulosa cells (GC), and antrum (An) of the tertiary follicle (Ter) (**i**–**l**).* White arrows* point to similar localization of CD63 tetraspanin (*green*) and alpha V integrin (*red*) in granulosa cells (GC) of primordial follicles (Prim), the primary follicle (PR), the secondary follicle (Sec), and surrounding ovarian stroma (OS) (**a**'–**d**'), and in granulosa cells (GC), and antrum (An) of the tertiary follicle (Ter) (**e**'–**h**').* Yellow arrows* refer to the follicle stage. Tetraspanin CD9, CD63 (*green*), alpha V integrin (*red*), DNA (*blue*). The* scale bar* represents 50 µm

The double fluorescent assay showed similar localization of tetraspanins CD9 and CD63, and integrin alpha V. The examined proteins were observed in granulosa cells of primordial follicles as well as primary follicles, and secondary follicles (Fig. 8e–h, Fig. 8a'–d'), and also in surrounding ovarian stroma (Fig. 8a–h, Fig. 8 a'–d'). In the tertiary follicle, both tetraspanins and integrin alpha V were localized in theca cells, granulosa cells, and antrum (Fig. 8i–l, Fig. 8 e'–h').

**Fig. 9:**
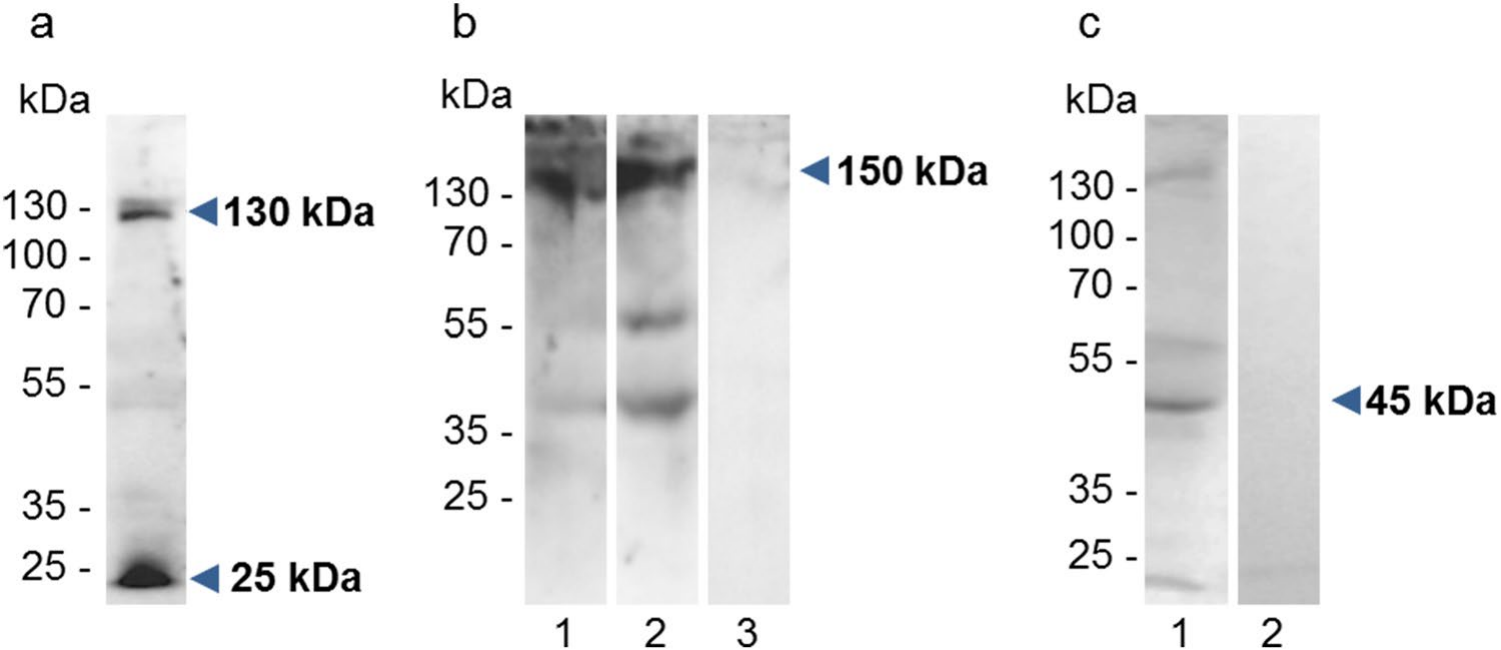
Interaction of CD9 and CD63 tetraspanins with αV integrin. Immunodetection of αV integrin in ovarian tissue lysates under reducing conditions, anti-αV antibody detected bands in molecular mass of ∼ 130 and ∼ 25 kDa (*blue arrows*) corresponding to the heavy and light chain of αV integrin (**a**). Detection of αV integrin in immunoprecipitates of CD9 (lane 1), and CD63 (lane 2), band with a molecular mass of ∼150 kDa (*blue arrow*). Other bands probably represent the heavy and light chain of immunoglobulin. As a negative control, immunoprecipitation with mouse IgG1 antibody is shown in lane 3 (**b**). Detection of CD63 tetraspanin in the immunoprecipitate of αVβ3 antibody, band with the molecular mass of ∼ 45 kDa (*blue arrow*). Immunoprecipitation with mouse IgG1 antibody as a negative control is shown in lane 2 (**c**)

To verify a possible interaction of tetraspanins with integrin alpha V, we performed the co-immunoprecipitation analysis of ovarian tissue lysate. In precipitates with monoclonal anti-CD9 and anti-CD63 antibodies, we detected αV integrin in the molecular mass of ∼130 kDa (Fig. 9b). In addition, the CD63 antibody reciprocally recognized the band with the molecular mass of ∼45 kDa in immunoprecipitate with the anti-αVβ3 monoclonal antibody (Fig. 9c).

### Presence of tetraspanins CD151, CD82, CD63, CD9, and CD81 in bovine fresh (immature and mature) and vitrified mature oocytes

**Fig. 10:**
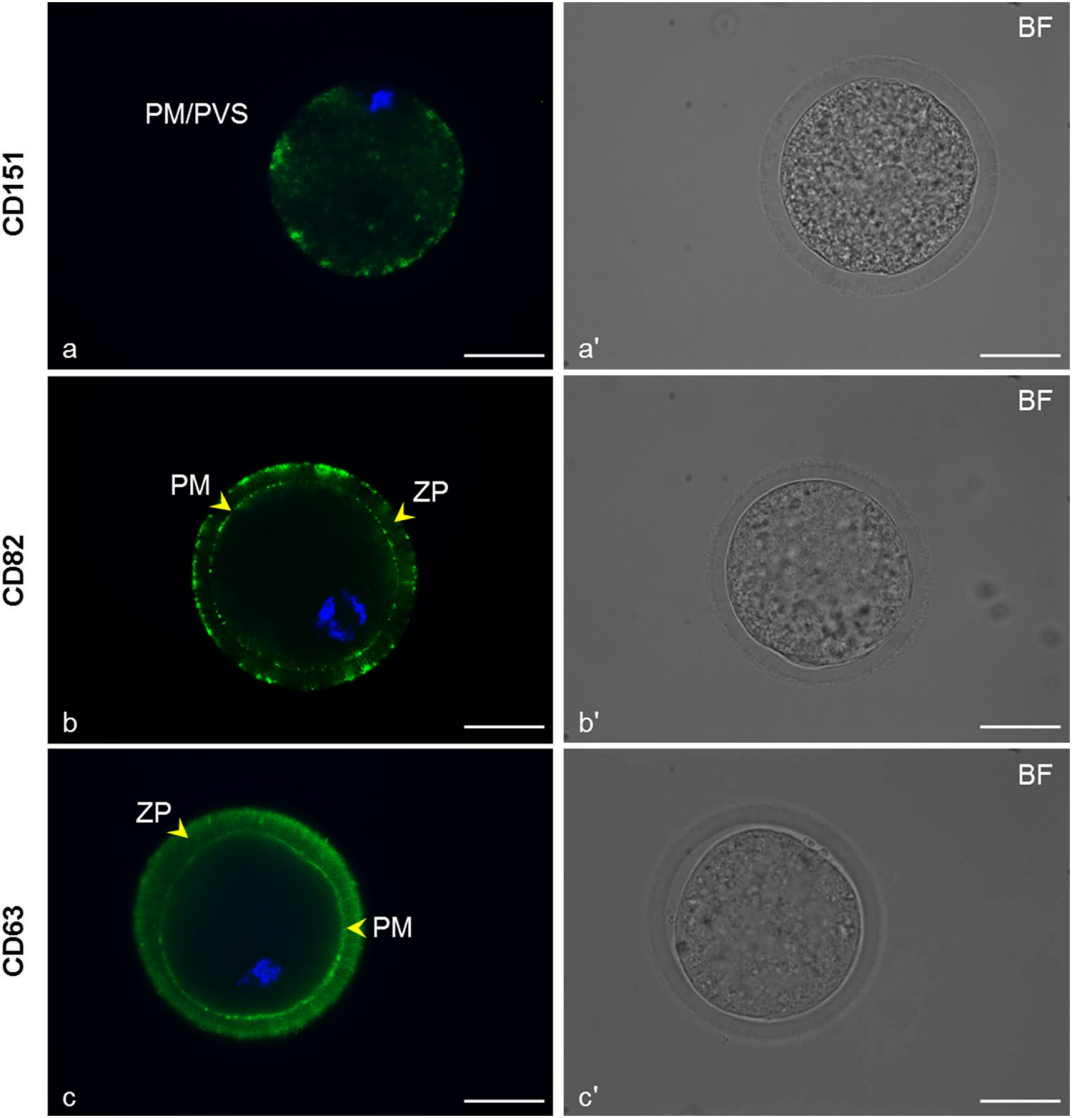
Localization of tetraspanins CD151, CD82, and CD63 in fresh immature cow oocytes.* Arrows* point to CD151 (*green*) localization in clusters along the plasma membrane (PM) and/or in the perivitelline space (PVS) (**a**), CD82 (*green*) detection in clusters near the PM and *zona pellucida* (ZP) (*arrows*) (**b**), CD63 (*green*) detection in clusters near the PM and ZP (*arrows*) (**c**). DNA (*blue*). Images marked by* letters with an apostrophe* represent the bright field images (BF) corresponding to the fluorescence image marked with the same letter. The* scale bar* represents 50 µm

**Fig. 11:**
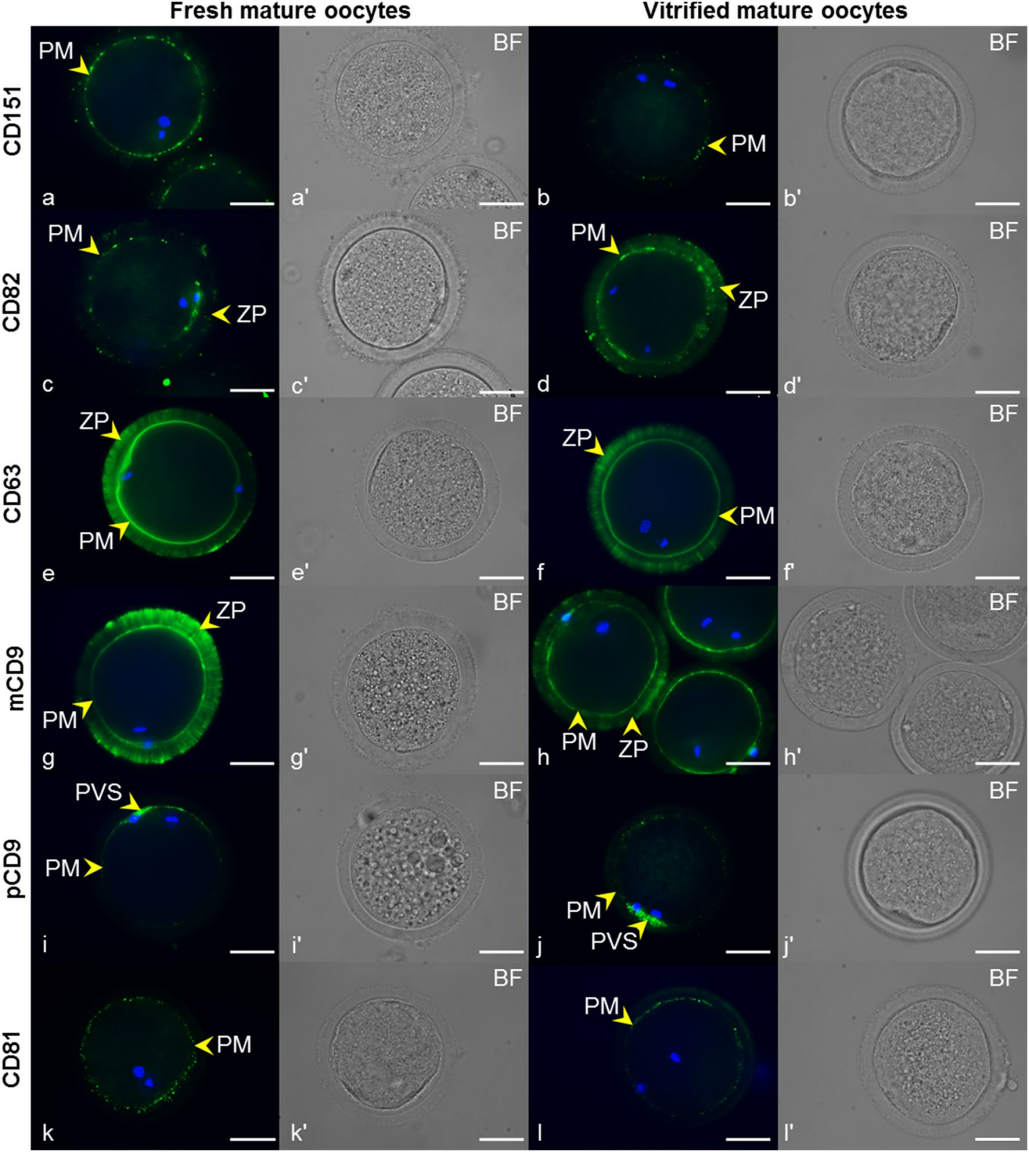
Localization of tetraspanins CD151, CD82, CD63, CD9, and CD81 in fresh and vitrified mature cow oocytes.* Arrows* show CD151 (*green*) localized in clusters along the plasma membrane (PM) in fresh mature (**a**) and vitrified mature oocytes (**b**), CD82 (*green*) detection in clusters near the PM and *zona pellucida* (ZP) in fresh mature (**c**) and vitrified mature oocytes (**d**), CD63 (*green*) localized to the PM and ZP of fresh mature (**e**) and vitrified mature oocytes (**f**), CD9 localization, detected by a monoclonal antibody, mCD9 (*green*), in PM and ZP in fresh mature (**g**) and vitrified mature oocytes (**h**). Detection of CD9 using a polyclonal antibody, pCD9 (*green*) in the PM with extension to the perivitelline space (PVS) in fresh mature (**i**) and vitrified mature oocytes (**j**), CD81 localization (*green*) in clusters in the PM of fresh mature (**k**) and vitrified mature oocytes (**l**). DNA (*blue*). Images marked by* letters with an apostrophe* represent the bright-field images (BF) corresponding to their respective fluorescence image. The* scale bar* represents 50 µm

**Fig. 12:**
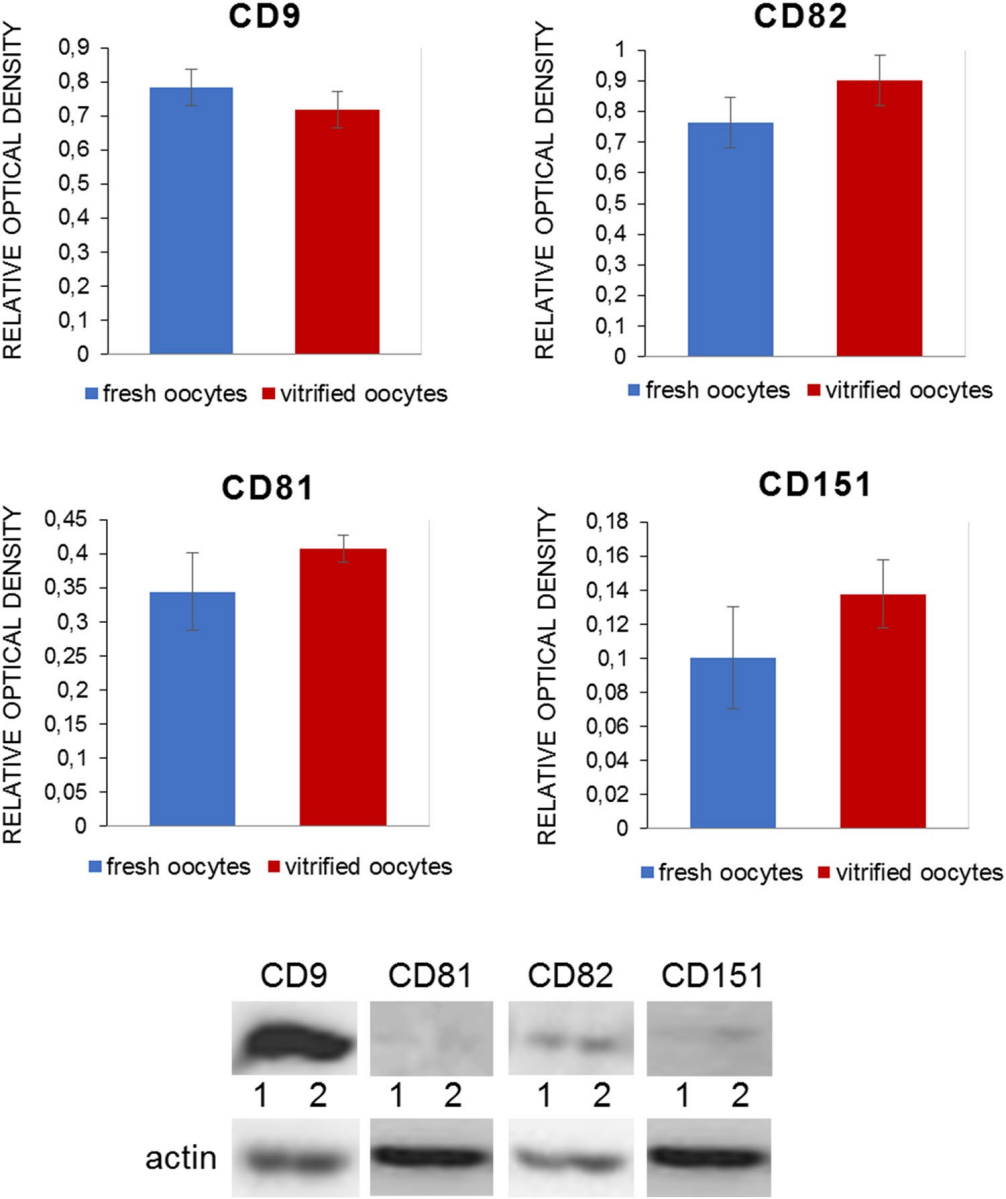
The relative optical density of CD9, CD81, CD82, and CD151 in bovine oocytes. Representative images of Western-blot analysis for targeted tetraspanins in bovine fresh (lane 1) and vitrified oocytes (lane 2). The relative density of individual tetraspanins was calculated as the ratio of the optical density of tetraspanins and actin antibodies signal in the blot.* Bars* represent the mean ± SEM of the density ratio of CD9, CD81, CD82, and CD151 to actin of three replicates. No statistically significant changes in protein levels in fresh vs. vitrified oocytes were revealed

By immunofluorescent assay, tetraspanin CD151 was detected in visible clusters lining up along the plasma membrane and/or in the perivitelline space of immature oocytes (Fig. 10a). In mature (MII) oocytes, CD151 was observed in the clear clusters appertained to the plasma membrane (Fig. 11a). The localization of this tetraspanin did not change in vitrified oocytes compared with the fresh mature oocytes (Fig. 11b). Detected reaction patterns of tetraspanins CD82 and CD63 on immature cells (Fig. 10b, c) and mature (Fig. 11c, e) oocytes appear to be very similar. In addition to the intensively stained clusters of both tetraspanins localized in or near the plasma membrane, filaments overlapping the *zona pellucida* and strongly resembling transzonal projections were detected. We also noted the CD82 signal on the outer margin of the ZP, probably from cumulus cells, which were also positively stained. In the vitrified oocytes, no significant change in the distribution of tetraspanins CD82 or CD63 was observable (Fig. 11d, f). The vitrified mature oocytes did not show an altered localization of tetraspanins CD9 and CD81 in comparison with the localization in the fresh immature and mature oocytes documented in our previous studies (Jankovicova et al. 2016, 2019). Fresh mature oocytes were examined as a control sample in this study. Monoclonal anti-CD9 antibody stained the plasma membrane homogenously, moreover, we detected CD9 positive filament-like structures passing through the *zona pellucida* (Fig. 11g, h). However, a polyclonal antiCD9 antibody recognized CD9 in the interrupted line on the plasma membrane with the partial extension of the CD9 clusters into the perivitelline space (Fig. 11i, j). In the case of CD81, clusters located along the plasma membrane were detected, but there was no specific signal in the ZP (Fig. 11k, l). The results are summarized in Table 2. Negative controls are shown in Supplementary Fig. 3, 4. Densitometric analysis of CD9, CD81, CD82, and CD151 Western blots did not reveal any statistically significant changes in protein level in fresh vs. vitrified oocytes (Fig. 12). CD63 antibody failed to detect protein bands in oocyte lysates.

### In silico analysis of CD9, CD81, CD151, CD63, and CD82 transcripts with expression in ovaries and oocytes

In silico analysis was performed using the Bgee database (release 14.2) and Ensembl database (release 104—May 2021). The obtained data addressing cow ovaries and oocytes are summarized in Table 3 (*Bos taurus*). The data concerning the other species are presented in Supplementary Tables: Table S1 (*Homo sapiens*), Table S2 (*Sus scrofa*), Table S3 (*Mus musculus*) and Table S4 (summarized data).

**Table 3:** Summary of in silico analysis of CD9, CD81, CD151, CD63, and CD82 transcripts with expression in cow ovary (*Bos taurus*)

CD9						
Transcript	UniProt match	Gene	Expression	Rank score	Expression score	Sources
ENSBTAT00000019643.5	P30932	ENSBTAG00000014764	Granulosa cells	2.81e3	87.83	R
			Theca cells	765	96.69	R
			Cumulus cells	5.92e3	74.35	R

CD81						
Transcript	UniProt match	Gene	Expression	Rank score	Expression score	Sources
ENSBTAT0000006558	Q3ZCD0	ENSBTAG00000047495	Granulosa cells	403	98.26	R
			Theca cells	232	99.00	R
			Cumulus cells	1.84e3	92.04	R

CD151						
Transcript	UniProt match	Gene	Expression	Rank score	Expression score	Sources
ENSBTAT00000053083.2	A7E3T1	ENSBTAG00000019569	Granulosa cells	350	98.49	R
			Theca cells	255	98.90	R
			Cumulus cells	1.25e3	94.61	R

CD63						
Transcript	UniProt match	Gene	Expression	Rank score	Expression score	Sources
ENSBTAT00000015829.4	B0JYM4 Q9XSK2	ENSBTAG00000011931	Granulosa cells	157	99.33	R
			Theca cells	145	99.37	R
			Cumulus cells	519	97.76	R

CD82						
Transcript	UniProt match	Gene	Expression	Rank score	Expression score	Sources
ENSBTAT00000078576.1	A5D7E6	ENSBTAG00000031252	Granulosa cells	4.71e3	79.60	R
			Theca cells	728	96.85	R
			Cumulus cells	3.11e3	86.53	R

Transcript IDs and Genes are listed in Ensembl ID format (https://www.ensembl.org/). UniProt Match refers to the UniProt identifier of protein (https://www.uniprot.org/) that corresponds to the Ensembl transcript. Only transcripts encoding the protein were included. Expression data were retrieved from the Bgee database (release 14.2) (http://bgee.org/). Rank scores of expression calls are normalized across genes, conditions, and species. A low score means that the gene is highly expressed in the condition. Max rank score in all species: 4.10e4. Min rank score varies across species. Expression scores of expression calls use the minimum and maximum Rank of the species to normalize the expression to a value between 0 and 100. A low score means that the gene is lowly expressed in the condition. Sources of data: R-RNA-Seq. Not listed cell types and tissues have not been tested

## Discussion

Despite the critical role of tetraspanin proteins in many essential physiological processes of mammalian cells, the distribution of CD9, CD81, CD151, CD82, and CD63 in the bovine ovary has not yet been described. Compared to the other species, our results are in agreement with Takao et al. (1999) who referred CD9 expression in the human follicular epithelium through all stages of follicle development and with findings of Li et al. (2004), where CD9 staining was observed on granulosa cells and the oocyte plasma membrane in preantral and fully grown follicles in porcine ovaries. We also observed positive staining in the area of the developing oocytes and their proximity as early as in the primordial and primary follicles. In later stages of follicle development, the basement membrane and ovarian stroma were also positively stained (Fig. 2, 3). In mice, CD9 was observed on some theca layer cells, while the surrounding ovarian tissue was negative (Chen et al. 1999). The presence of CD9 was also confirmed in immature (Chen et al. 1999) and mature mouse oocytes (Le Naour et al. 2000). Consistent with our findings (Fig. 4), the immunohistochemical analysis of the adult wild-type mouse ovaries showed the continuous expression of CD81 in the egg and surrounding follicles as well as in cumulus cells around the ovulated eggs (Tanigawa et al. 2008). Tetraspanin CD151 was observed on human ovarian epithelial cells, where it was primarily expressed on the membrane surface (Mosig et al. 2012). Regarding CD63 and CD82 tetraspanins, no immunofluorescent analysis concerning the expression, localization, or function in mammalian ovaries is available for comparison with our results (Figs. 6, 7).

The tetraspanins CD9, CD81, CD82, and CD151 were detected by Western-blot analysis of ovarian protein extract as the bands at molecular weight ~ 22–24 kDa with additional bands that most likely correspond to their dimers (Kovalenko et al. 2004). The broad signal in the range from 31 to 45 kDa detected for CD63 probably reflects the highly glycosylated form of protein (UniProt Consortium 2021).

Tetraspanins generally act within the tetraspanin web, a functional multi-molecular complex composed of different tetraspanins and their interacting partner molecules (Zuidscherwoude et al. 2015). In cell membranes, including those of gametes, tetraspanins usually form complexes with integrins (Maecker et al. 1997; Berditchevski and Odintsova 1999; Stipp 2010; Merc et al. 2021), whose functions also regulate (Hemler 2005). Integrins are the adhesion receptors that act as transmembrane linkers between the extracellular matrix and the actin cytoskeleton, with the ability to transmit outside-in and vice versa signaling (Longhurst and Jennings 1998). Based on the study by Yu et al. (2017) performed on Chinese hamster, ovary cells expressed human integrins where αVβ3 was revealed as a receptor for tetraspanins CD9, CD81, and CD151 that directly binds to their large extracellular domain, we examined the localization of integrin subunit alpha V in cow ovary to identify it as the potential partner of tetraspanins using a combination of mouse monoclonal antibodies (CD9, CD63) and rabbit polyclonal antibody (αV). The double fluorescent assay has shown the distribution of CD9, CD63 and alpha V integrin in similar areas of ovarian tissue (Fig. 8). Additionally, based on co-immunoprecipitation analysis of ovarian tissue, we suggested the possible interaction of CD9 and CD63 with integrin alpha V. The signal appertaining to alpha V integrin and tetraspanin CD9 and CD63 (and also CD81, CD151, CD82) were observed in the follicle antrum, which suggests they are all the parts of extracellular vesicles released to this area. Assuming that the spatial proximity of proteins is a prerequisite for their cooperation, the participation of studied tetraspanins in molecular pathways involved in the morphological and functional changes of cells during folliculogenesis and oogenesis could be suggested although the confirmation needs further exploration. Tetraspanin CD151 directly associates with laminin-binding integrins and via them with other tetraspanin and non-tetraspanin partners (Stipp 2010). Laminins (and integrins) are considered to be crucial extracellular matrix regulators playing a notable role in tissue morphogenesis and homeostasis, cell adhesion, migration, and matrix-mediated signaling processes (reviewed in Longhurst and Jennings 1998; Tzu and Marinkovich 2008; Hamill et al. 2009). All of the processes also take place in the ovary. It was proposed that the extracellular matrix participates in the regulation of oocyte quality during ovulation (Honda et al. 2004). Laminin's role was linked to the modulation of ovine granulosa cell functions (through integrin α6β1) (Le Bellego et al. 2002), and a correlation of laminin concentrations in human follicular fluid with granulosa cell luteinization and oocyte quality was found (Honda et al. 2004). Because laminins are a major component of the basement membrane, it is interesting that we observed not only CD151 but also CD9, CD81, CD63, and CD82 in this area of bovine follicles (Figs. 2, 3, 4, 5, 6, 7). In the context of integrins, it is appropriate to note that the responsibility of CD81 for the connection of CD19 to the tetraspanin web and associated molecules such as integrins was suggested (Horváth et al. 1998). CD63 was confirmed to directly interact with syntenin-1, a double PDZ domain-containing protein, which is involved in pathways that control cytoskeletal dynamics, vesicular trafficking, cell adhesion, and cell polarity (reviewed in Latysheva et al. 2006). Israels and McMillan-Ward (2010) referred that on activated platelet membranes, CD63 associates with the cytoskeleton only through its interaction with the CD9-αIIbβ3 complex. According to Delaguillaumie et al. (2004), another tetraspanin CD82 establishes a functional coupling of the raft domains and actin cytoskeleton. Recently, the requirement of CD82 interaction with cholesterol for induction of the extracellular release of ezrin by microvesicles was reported (Huang et al. 2020). In silico analysis of CD9, CD81, CD151, CD63, and CD82 transcripts in cow ovary revealed their expression in theca cells, granulosa cells, and cumulus cells. However, the germinal epithelium of the ovary, as well as the ovary as a whole, and oocytes have not yet been tested. In silico analysis did not provide complete data for comparison with humans, pigs, and mice, as most ovarian cell types were not tested. On the other hand, interspecies differences in oocyte expression of studied tetraspanins were revealed, which could indicate the unique species-dependent mechanisms of tetraspanins involvement. In our experiments, for tetraspanins CD81, CD151, CD82, and CD63, we observed at different stages of follicular development CD9-like distribution patterns. Considering in silico analysis and our results showing the presence of all analyzed tetraspanins in the follicle environment and the oocyte area from the first stages of development, it can be hypothesized that they play a role during oocyte and follicle development through the tetraspanin network, either in a partnership or individually. In particular, tetraspanins may be involved in the morphogenesis of follicles and granulosa cells or the organization of the cell membrane, presumably through association with integrins via the cytoskeleton. In the line with the ability of tetraspanins to form a scaffold for signaling pathways, these tetraspanins could also participate in molecular trafficking via extracellular vesicles (Andreu and Yáñez-Mó 2014). The molecular mechanisms by which tetraspanins participate in follicle functions, oocyte development, and fertilization remain unclear, and moreover, the species-specific traits also supported by in silico analysis should be considered.

In our previous studies, we reported the localization of two tetraspanins, CD9 and CD81, in the plasma membrane and PVS of pig and bovine oocytes and the ZP of bovine oocytes (Jankovicova et al. 2016, 2019). We suggested that while in the plasma membrane they more likely participate in membrane organization and curvature (Frolikova et al. 2018), the detected presence of CD9 in the *zona pellucida* of bovine oocytes could reflect a different role at least of this tetraspanin, that is probably related to processes associated with cell–cell communication. In the presented study, we documented a similar reaction pattern of CD63 and CD82 tetraspanins in ZP. These observations agree with our previous findings of CD9 in filament-like structures resembling TZPs in the *zona pellucida* of immature and mature bovine oocytes (Jankovicova et al. 2019). Recognizable signals of CD63 and CD9 in the ZP were also observed in the present study in oocytes within mature follicles. Transzonal projections can be characterized as actin-rich filaments that project from somatic granulosa cells, penetrate the *zona pellucida*, and contact the oocyte plasma membrane (Clarke 2018). The main role of TZPs is considered to be the mediation of the bilateral communication between the growing oocyte and the follicle environment. Small molecules such as ions, amino acids, sugars, energy substrates, and cyclic nucleotides as well as lipids, small organelles, and even mRNAs can be transported through transzonal projections (reviewed in Macaulay et al. 2014, 2016; Russell et al. 2016; Andrade et al. 2019). Furthermore, this communication is mediated not only by TZPs molecular transport and secretion of paracrine factors but also possibly by extracellular vesicles (EVs) (Macaulay et al. 2016; da Silveira et al. 2017; del Collado et al. 2017). It is generally accepted that extracellular vesicles act as mediators of intercellular communication (Le Naour et al. 2000; Miyado et al. 2000; Kaji et al. 2000) and that tetraspanins are usually used as markers of extracellular vesicles. CD63-, CD9-, and CD81-positive extracellular vesicles were observed in follicular and oviductal fluid, oocytes, and embryos (reviewed in Jankovičová et al. 2020b). Da Silveira et al. (2012) identified extracellular vesicles isolated from bovine follicular fluid in TZPs and demonstrated their uptake by granulosa and cumulus cells. The same authors documented extracellular vesicle uptake from the equine follicular fluid by surrounding granulosa cells. CD63-positive EVs were found in the follicular fluid of several mammals (equine (da Silveira et al. 2012), porcine (Matsuno et al. 2019), and human (Santonocito et al. 2014; Hu et al. 2020)), including bovines (Sohel et al. 2013), and their role in the regulation of follicle and/or embryo development was suggested (da Silveira et al. 2014). Hung et al. (2015) reported the ability of CD81-enriched EVs retrieved from small bovine follicles to upregulate cumulus gene expression in vitro and stimulate cumulus expansion. Therefore, in accordance with our previous results (Jankovicova et al. 2019), we hypothesize that the specific reaction pattern of CD63 and CD82 in the ZP might point to the presence of tetraspanin-positive vesicles in transzonal projections. Since no data regarding the regulatory mechanisms of molecular transport via extracellular vesicles by TZPs are currently available, we speculate that tetraspanins might be involved in this phenomenon. In contrast to CD63 and CD82, tetraspanin CD151 was organized in clusters along the plasma membrane and/or in the perivitelline space of immature as well as mature oocytes. In the study of Ziyyat et al. (2006), CD151 and CD9 together with integrin α6β1 were found to be evenly distributed on human zona-intact oocytes. However, in human ZP-free oocytes, the tetraspanins CD151 and CD81 were colocalized with integrin α6β1 in patches on the plasma membrane surface. These authors even assumed that α6β1 and CD151 participate in the control of human gamete fusion. Interestingly, a species-specific pattern of known tetraspanin partners, integrins have been detected in porcine and bovine oocytes (Linfor and Berger 2000; Pate et al. 2007). Given the literature data, our previous results regarding CD9 and CD81 (Jankovicova et al. 2016, 2019) and the presence of CD151, CD63, and CD82 in clusters lining up along the plasma membrane, the involvement of tetraspanins, potentially via -integrin complexes, in the establishment of the sperm-oocyte interaction machinery and/or other bovine fertilization events could be considered. We assume that tetraspanin localization determines their interaction partners and specific role within the tetraspanin web. Tetraspanin complexes were proposed to associate with lipid rafts (Israels and McMillan-Ward 2007; Xu et al. 2009; Huang et al. 2020). Defects in lipid raft microdomains have been implicated in the failure of human fertilization at the oocyte plasma membrane level (Van Blerkom and Caltrider 2013). It was evidenced in mice that membrane raft integrity is essential to complete fertilization (Buschiazzo et al. 2013). In the plasma membrane of vitrified bovine oocytes, the changed level as well as localization of GM1 (monosialotetrahexosylganglioside), a lipid rafts marker was detected (Simons and Ikonen 1997; Buschiazzo et al. 2017). We assumed that vitrification can disturb the oocyte plasma membrane organization required for fertilization, and subsequently affect tetraspanin distribution. A changed distribution pattern of CD9 on the bovine oocyte plasma membrane was documented by Zhou et al. (2013), in contrast to the results reported by Wen et al. (2007) on mouse oocytes. Our immunofluorescent and Western-blot analysis did not reveal changes in localization and protein levels of CD9, CD81, CD151, CD82, and CD63 on vitrified oocytes. However, to analyze potential alterations in tetraspanin distribution after oocyte vitrification in detail, further analysis is needed.

## Conclusions

The present study brings new knowledge regarding the localization profiles of the tetraspanins CD9, CD81, CD151, CD82, and CD63 in cattle ovarian follicles. Tetraspanins were found in the follicular epithelium through all stages of follicle development. Moreover, a distribution pattern for CD151, CD82, and CD63 in immature and mature bovine oocytes was detected. Notably, the presence of CD63 and CD82 in the *zona pellucida* was also recorded. Furthermore, no changes were observed in the distribution patterns of CD9, CD81, CD151, CD82, and CD63 tetraspanins in vitrified mature oocytes. The study also provides in silico analysis that suggested the differences in tetraspanins expression in the ovarian cells and oocytes across species, such as cattle, humans, pigs, and mice. The obtained results suggest that in the study of oocyte development and potentially also the fertilization process of cattle, the role of tetraspanins, and integrins should also be taken into account.

## Supplementary Information

Below is the link to the electronic supplementary material.

Supplementary file1 (DOCX 3515 KB)
