# Supplementary material for: Distribution of tetraspanins in bovine ovarian tissue and fresh/vitrified oocytes
Source: Histochem Cell Biol. 2022 Oct 15;159(2):163–83. doi: 10.1007/s00418-022-02155-4 (PMC9922244; doi:10.1007/s00418-022-02155-4)
Supplement: Supplementary file 1 — Supplementary file1 (DOCX 3515 KB) [file 418_2022_2155_MOESM1_ESM.docx]

**Distribution of tetraspanins in bovine ovarian tissue**

**and fresh/vitrified oocytes**

**Jana Jankovičová^1*^, Petra Sečová^1^, Ľubica Horovská^1^, Lucia Olexiková^2^, Linda Dujíčková^2,3^, Alexander V. Makarevich^2^, Katarína Michalková^1^ and Jana Antalíková^1^**

^1^Laboratory of Reproductive Physiology, Institute of Animal Biochemistry and Genetics, Centre of Biosciences, Slovak Academy of Sciences, Bratislava, Slovak Republic

^2^National Agricultural and Food Centre, Research Institute for Animal Production Nitra, Lužianky, Slovak Republic

^3^ Department of Botany & Genetics, Faculty of Natural Sciences and Informatics, Constantine the Philosopher University in Nitra, Slovak Republic

**^*^**Corresponding author: Dr. Jana Jankovičová, Institute of Animal Biochemistry and Genetics, Centre of Biosciences, Slovak Academy of Sciences, Dúbravská cesta 9, 84005 Bratislava, Slovak Republic

E-mail: [jana.jankovicova@savba.sk](mailto:jana.jankovicova@savba.sk)

ORCID: Jana Jankovičová 0000-0002-4714-6184

| **Supplementary information content:** |  |
| --- | --- |
| Supplementary Fig. 1 .................................................................................................................. | S2 |
| Supplementary Fig. 2 .................................................................................................................. | S3 |
| Supplementary Fig. 3 .................................................................................................................. | S4 |
| Supplementary Fig. 4 .................................................................................................................. | S5 |
| Supplementary Table 1 ............................................................................................................... | S6 |
| Supplementary Table 2 ............................................................................................................... | S9 |
| Supplementary Table 3 ............................................................................................................... | S10 |
| Supplementary Table 4 ............................................................................................................... | S12 |

| 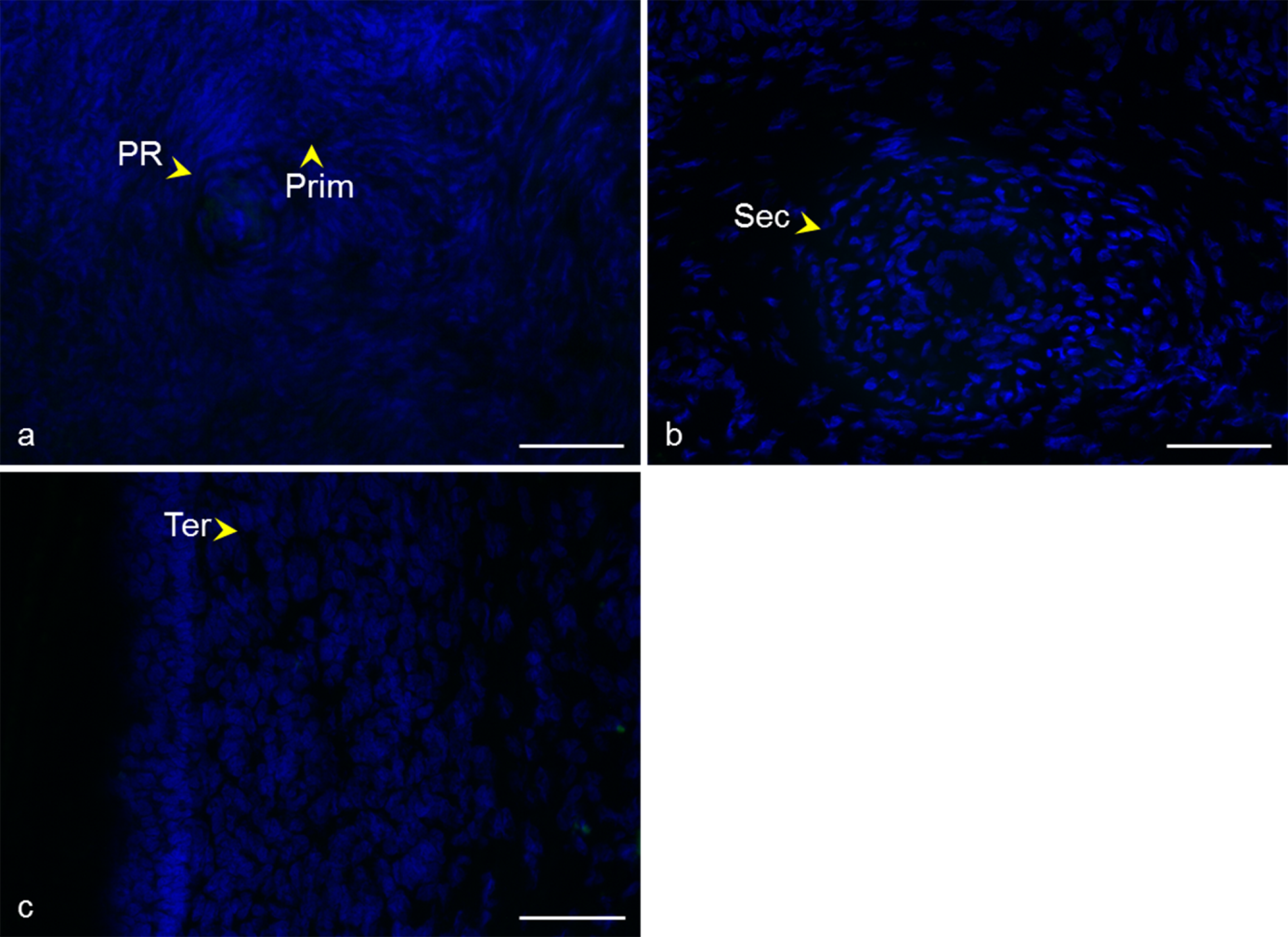 |  |
| --- | --- |

**Supplementary Fig. 1** Negative control for CD9 and CD63 tetraspanins detected by monoclonal antibodies. Primordial follicle (Prim) (**a**), primary follicle (PR) (**a**), secondary follicle (Sec) (**b**), tertiary follicle (Ter) (**c**). DNA (blue), isotype control (green). The scale bar represents 50 µm

**
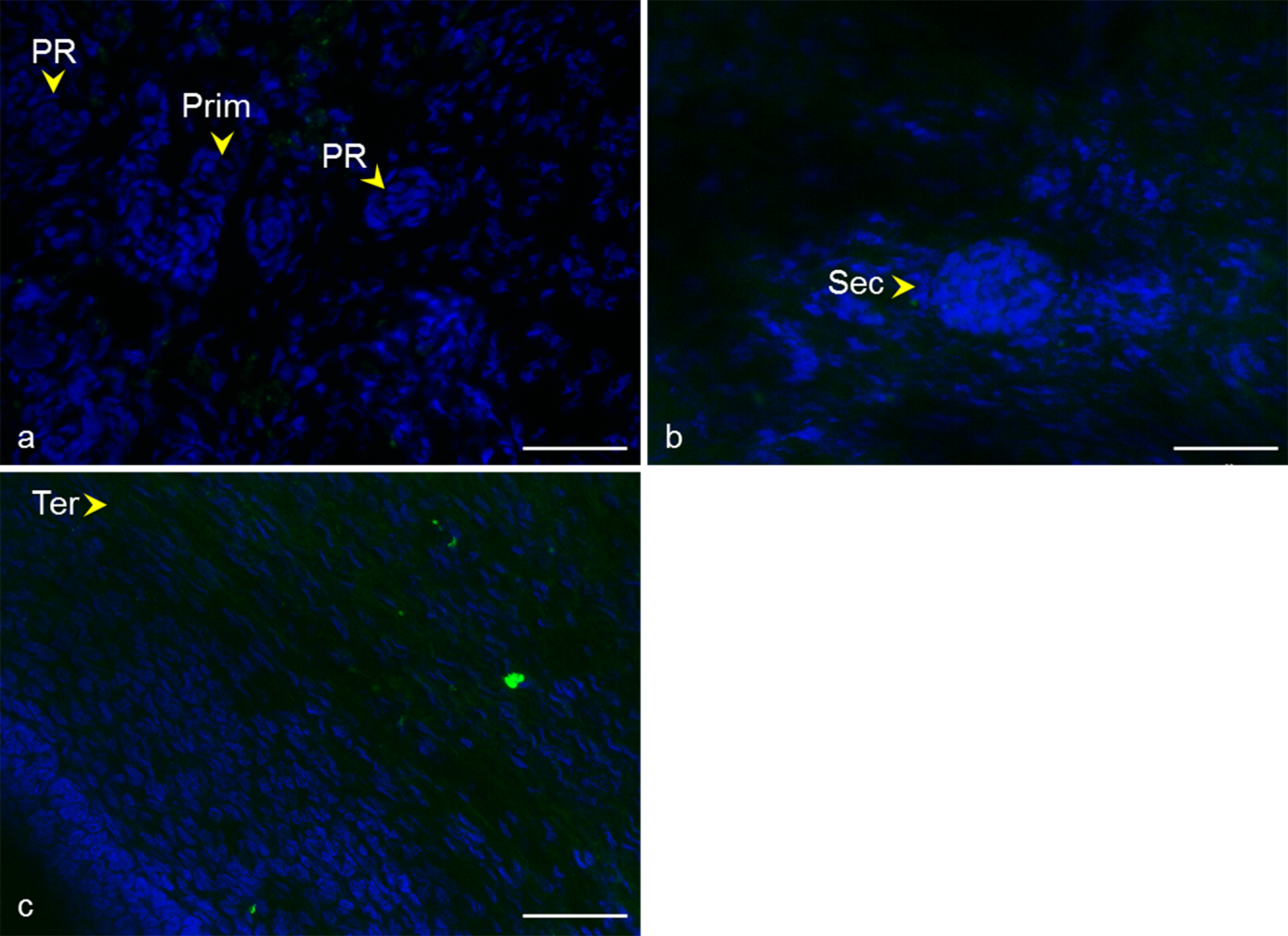
**

**Supplementary Fig. 2** Negative control for tetraspanins CD9, CD81, CD151 and CD82 detected by polyclonal antibodies. Primordial follicle (Prim) (**a**), primary follicle (PR) (**a**), secondary follicle (Sec) (**b**), tertiary follicle (Ter) (**c**). DNA (blue), isotype control (green). The scale bar represents 50 µm

**
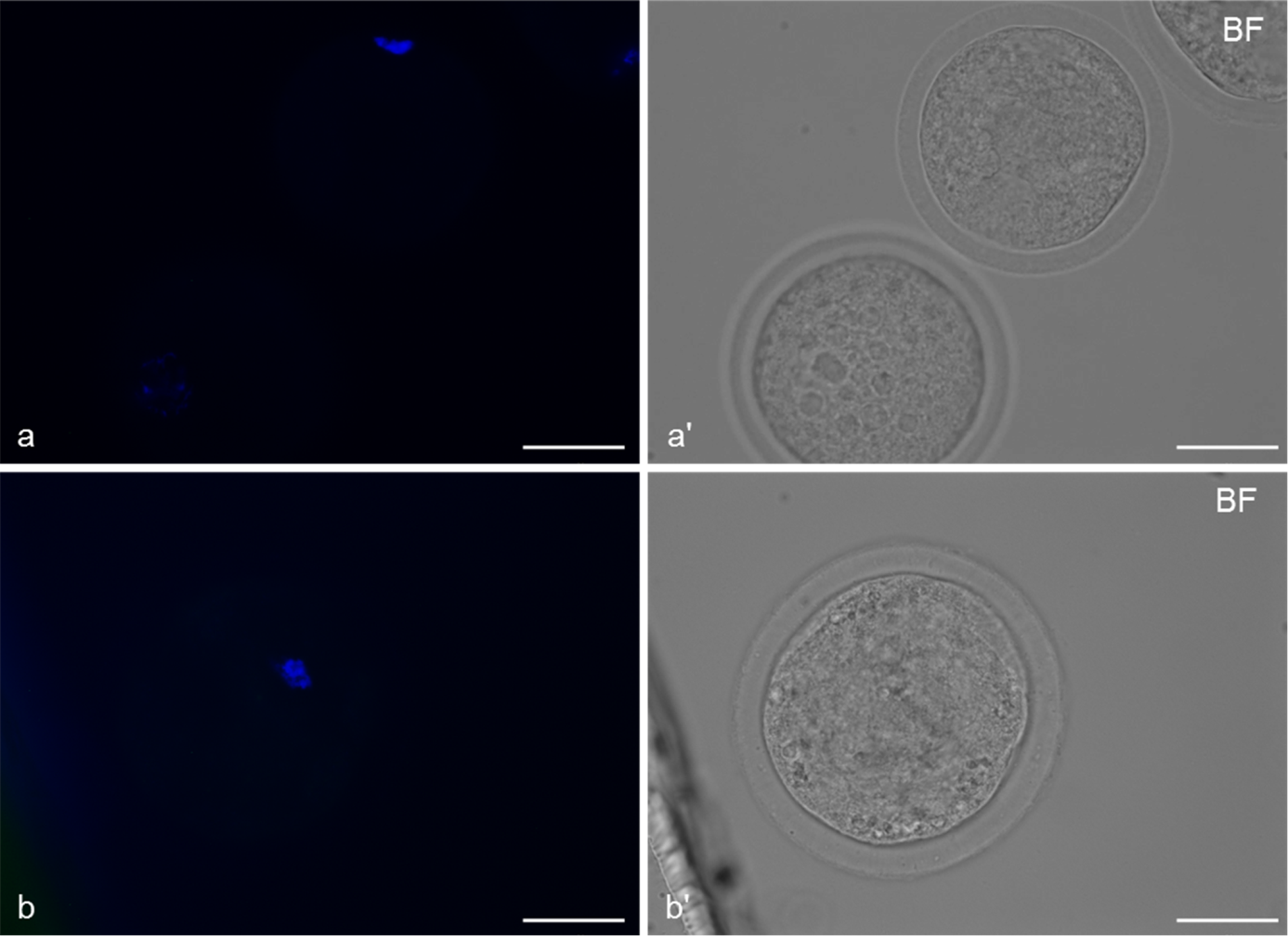
**

**Supplementary Fig. 3** Negative control for CD151, CD82 detected by polyclonal antibodies in fresh immature oocytes (**a**). Negative control for CD63 detected by monoclonal antibody in fresh immature oocytes (**b**). DNA (blue), isotype control (green). Images marked by letters with an apostrophe represent the bright field images (BF) corresponding to their respective fluorescence image. The scale bar represents 50 µm

**
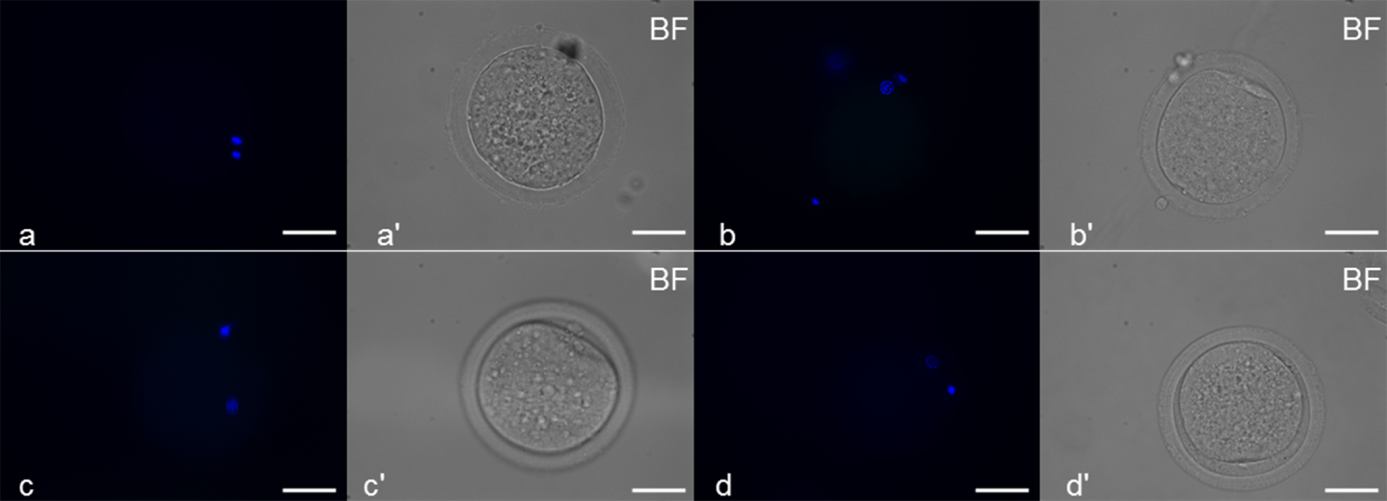
**

**Supplementary Fig. 4** Negative control for CD9 and CD63 tetraspanins detected by monoclonal antibodies in fresh mature (**a**) and vitrified mature oocytes (**b**). DNA (blue), isotype control (green). Negative control for CD9, CD81, CD151 and CD82 tetraspanins detected by polyclonal antibodies in fresh mature (**c**) and vitrified mature oocytes (**d**). DNA (blue), isotype control (green). Images marked by letters with an apostrophe represent the bright field images (BF) corresponding to their respective fluorescence image. The scale bar represents 50 µm

**Supplementary Table 1 Summary of in silico analysis of CD9, CD81, CD151, CD63 and CD82 transcripts with expression in human (*Homo sapiens*) ovary and oocytes**

| **CD9** | | | | | | |
| --- | --- | --- | --- | --- | --- | --- |
| **Transcript** | **UniProt Match** | **Gene** | **Expression** | **Rank score** | **Expression score** | **Sources** |
| ENST00000009180.10 | P21926 | ENSG00000010278 | Germinal epithelium of the ovary  Right ovary  Left ovary  Secondary oocyte  Oocyte | 503  1.87e3  2.39e3  4.76e3  7.46e3 | 99.09  96.60  95.67  91.36  86.45 | A  R  R  A  A |
| ENST00000382518.6 | P21926 |  |  |  |  |  |
| ENST00000538834.6 | P21926 |  |  |  |  |  |
| ENST00000642746.1 | A6NNI4 |  |  |  |  |  |
| ENST00000382515.7 | A6NNI4 |  |  |  |  |  |
| ENST00000646407.1 | A6NNI4 |  |  |  |  |  |
| ENST00000610354.5 | A0A087WU13 |  |  |  |  |  |
| ENST00000536586.7 | F5GXT1 |  |  |  |  |  |
| ENST00000382519.9 | G8JLH6 |  |  |  |  |  |
| **CD81** | | | | | | |
| **Transcript** | **UniProt Match** | **Gene** | **Expression** | **Rank score** | **Expression score** | **Sources** |
| ENST00000263645.10 | A0A024RCB7, P60033 | ENSG00000110651 | Right ovary  Left ovary  Germinal epithelium of the ovary  Oocyte  Secondary oocyte | 166  183  407  5.87e3  6.77e3 | 99.70  99.67  99.26  89.34  87.71 | R  R  A  A  A |
| ENST00000526072.5 | E9PJK1 |  |  |  |  |  |
| ENST00000492627.5 | E9PJK1 |  |  |  |  |  |
| ENST00000492252.5 | E9PRJ8 |  |  |  |  |  |
| ENST00000481687.1 | E9PIF1 |  |  |  |  |  |
| ENST00000381036.7 | A6NMH8 |  |  |  |  |  |
| ENST00000464784.6 | H0YDL9 |  |  |  |  |  |
| ENST00000527343.5 | H0YDJ9 |  |  |  |  |  |
| ENST00000475945.6 | E9PM31 |  |  |  |  |  |
| ENST00000530648.5 | E9PPF5 |  |  |  |  |  |
| ENST00000533417.5 | H0YEE2 |  |  |  |  |  |
| ENST00000493525.6 | E9PQV4 |  |  |  |  |  |
|  | | | | | | |
|  | | | | | | |
|  | | | | | | |
| **CD151** | | | | | | |
| **Transcript** | **UniProt Match** | **Gene** | **Expression** | **Rank score** | **Expression score** | **Sources** |
| ENST00000397420.9 | A0A024RCB3, P48509 | ENSG00000177697 | Right ovary  Left ovary  Germinal epithelium of the ovary  Oocyte | 631  746  8.75e3  absent | 98.86  98.65  84.12 | R  R  A |
| ENST00000322008.9 | A0A024RCB3, P48509 |  |  |  |  |  |
| ENST00000397421.5 | A0A024RCB3, P48509 |  |  |  |  |  |
| ENST00000530726.5 | A0A024RCB3, P48509 |  |  |  |  |  |
| ENST00000528011.2 | E9PMR4 |  |  |  |  |  |
| ENST00000527341.5 | K4DIB7 |  |  |  |  |  |
| ENST00000526693.5 | E9PLZ6 |  |  |  |  |  |
| ENST00000526439.1 | E9PP93 |  |  |  |  |  |
| ENST00000530320.5 | E9PJC8 |  |  |  |  |  |
| ENST00000529810.5 | E9PRJ3 |  |  |  |  |  |
| ENST00000525333.5 | E9PK37 |  |  |  |  |  |
| ENST00000528867.5 | E9PJE8 |  |  |  |  |  |
| ENST00000525718.6 | E9PSA1 |  |  |  |  |  |
| **CD63** | | | | | | |
| **Transcript** | **UniProt Match** | **Gene** | **Expression** | **Rank score** | **Expression score** | **Sources** |
| ENST00000257857.9 | P08962-1 | ENSG00000135404 | Right ovary  Left ovary  Germinal epithelium of the ovary  Oocyte  Secondary oocyte | 156  169  570  6.77e3  1.23e4 | 99.72  99.69  98.97  87.71  77.68 | R  R  A  A  A |
| ENST00000549117.5 | P08962-1 |  |  |  |  |  |
| ENST00000420846.7 | P08962-1 |  |  |  |  |  |
| ENST00000552692.5 | P08962-1 |  |  |  |  |  |
| ENST00000552754.5 | P08962-2 |  |  |  |  |  |
| ENST00000550776.5 | P08962-3 |  |  |  |  |  |
| ENST00000546939.5 | P08962-3 |  |  |  |  |  |
| ENST00000552164.5 | F8VWK8 |  |  |  |  |  |
| ENST00000551173.5 | F8W022 |  |  |  |  |  |
| ENST00000548898.5 | F8VV56 |  |  |  |  |  |
| ENST00000548160.5 | F8VV56 |  |  |  |  |  |
| ENST00000552067.5 | F8VV56 |  |  |  |  |  |
| ENST00000546457.1 | F8VNT9 |  |  |  |  |  |
| **CD82** | | | | | | |
| **Transcript** | **UniProt Match** | **Gene** | **Expression** | **Rank score** | **Expression score** | **Sources** |
| ENST00000227155.9 | P27701-1 | ENSG00000085117 | Right ovary  Left ovary  Germinal epithelium of the ovary  Oocyte  Secondary oocyte | 5.19e3  6.61e3  1.00e4  3.30e3  7.98e3 | 90.58  87.99  81.83  94.01  85.51 | R  R  A  A  A |
| ENST00000342935.7 | P27701-2 |  |  |  |  |  |
| ENST00000524750.1 | H0YD57 |  |  |  |  |  |
| ENST00000527737.5 | E9PM43 |  |  |  |  |  |
| ENST00000524704.5 | E9PP61 |  |  |  |  |  |
| ENST00000530601.1 | E9PJC7 |  |  |  |  |  |
| ENST00000532544.5 | E9PJK9 |  |  |  |  |  |
| ENST00000525813.5 | E9PJB7 |  |  |  |  |  |
| ENST00000526958.5 | E9PJ45 |  |  |  |  |  |
| ENST00000525210.5 | E9PJ59 |  |  |  |  |  |

**Transcript IDs and Genes** are listed in Ensembl ID format (<https://www.ensembl.org/>). **UniProt Match** refers to the UniProt identifier of protein (<https://www.uniprot.org/>) that corresponds to the Ensembl transcript. Only transcripts encoding the protein were included. **Expression** data were retrieved from the Bgee database (release 14.2) (<http://bgee.org/>). **Rank scores** of expression calls are normalized across genes, conditions and species. A low score means that the gene is highly expressed in the condition. Max rank score in all species: 4.10e4. Min rank score varies across species. **Expression scores** of expression calls use the minimum and maximum Rank of the species to normalize the expression to a value between 0 and 100. A low score means that the gene is lowly expressed in the condition. Sources of data: A-Affymetrix, R-RNA-Seq. Not listed cell types and tissues have not been tested.

**Supplementary Table 2 Summary of in silico analysis of CD9, CD81, CD151, CD63 and CD82 transcripts with expression in pig (*Sus scrofa*) ovary and oocytes**

| **CD9** | | | | | | |
| --- | --- | --- | --- | --- | --- | --- |
| **Transcript ID** | **UniProt Match** | **Gene** | **Expression** | **Rank score** | **Expression score** | **Sources** |
| ENSSSCT00000000773.4 | A0A4X1UUA2, F1SL24 | ENSSSCG00000022230 | Granulosa cells  Oocyte | 1.08e3  Absent | 95.41  - | R  - |
| **CD81** |  |  |  |  |  |  |
| **Transcript ID** | **UniProt Match** | **Gene** | **Expression** | **Rank score** | **Expression score** | **Sources** |
| ENSSSCT00000007005.5 | F1RJW5 | ENSSSCG00000006392 | Granulosa cells  Oocyte | 3.44e3  Absent | 85.43  - | R  - |
| **CD151** |  |  |  |  |  |  |
| **Transcript ID** | **UniProt Match** | **Gene** | **Expression** | **Rank score** | **Expression score** | **Sources** |
| ENSSSCT00000014035.4 | F1RYZ1 | ENSSSCG00000012840.5 | Granulosa cells  Oocyte | 5.43e3  984 | 76.99  95.83 | R  R |
| **CD63** |  |  |  |  |  |  |
| **Transcript ID** | **UniProt Match** | **Gene** | **Expression** | **Rank score** | **Expression score** | **Sources** |
| ENSSSCT00035032023.1 | A0A4X1W430, F1SPK8 | ENSSSCG00000000361 | Granulosa cells  Oocyte | 147  2.09e3 | 99.38  91.15 | R  R |
| ENSSSCT00000033233.3 | A0A4X1W430, F1SPK8 |  |  |  |  |  |
| **CD82** |  |  |  |  |  |  |
| **Transcript ID** | **UniProt Match** | **Gene** | **Expression** | **Rank score** | **Expression score** | **Sources** |
| ENSSSCT00000030279.4 | A0A4X1SFI7, B7ZEQ3 | ENSSSCG00000029521 | Granulosa cells  Oocyte | 2.95e3  Absent | 87.51  - | R  - |

**Transcript IDs and Genes** are listed in Ensembl ID format (<https://www.ensembl.org/>). **UniProt Match** refers to the UniProt identifier of protein (<https://www.uniprot.org/>) that corresponds to the Ensembl transcript. Only transcripts encoding the protein were included. **Expression** data were retrieved from Bgee database (release 14.2) (<http://bgee.org/>). **Rank scores** of expression calls are normalized across genes, conditions and species. Low score means that the gene is highly expressed in the condition. Max rank score in all species: 4.10e4. Min rank score varies across species. **Expression scores** of expression calls use the minimum and maximum Rank of the species to normalize the expression to a value between 0 and 100. Low score means that the gene is lowly expressed in the condition. Sources of data: A-Affymetrix, R-RNA-Seq. Not listed cell types and tissues have not been tested.

**Supplementary Table 3 Summary of in silico analysis of CD9, CD81, CD151, CD63, and CD82 transcripts with expression in mouse (*Mus musculus*) ovary and oocytes**

| **CD9** | | | | | | |
| --- | --- | --- | --- | --- | --- | --- |
| **Transcript** | **UniProt Match** | **Gene** | **Expression** | **Rank score** | **Expression score** | **Sources** |
| ENSMUST00000032492.9 | P40240 | ENSMUSG00000030342 | Primary oocyte  Secondary oocyte  Cumulus cells | 2.03e3  3.05e3  1.55e3 | 95.13  92.69  96.29 | A, R  A, R  A |
| **CD81** |  |  |  |  |  |  |
| **Transcript** | **UniProt Match** | **Gene** | **Expression** | **Rank score** | **Expression score** | **Sources** |
| ENSMUST00000037941.10 | P35762 | ENSMUSG00000037706 | Primary oocyte  Secondary oocyte  Cumulus cells | 1.11e4  9.71e3  317 | 73.43  76.70  99.24 | A, R  R  A |
| **CD151** |  |  |  |  |  |  |
| **Transcript** | **UniProt Match** | **Gene** | **Expression** | **Rank score** | **Expression score** | **Sources** |
| ENSMUST00000106000.10 | O35566 | ENSMUSG00000025510 | Primary oocyte  Secondary oocyte  Cumulus cells | 593  335  167 | 98.58  99.20  99.60 | A, R  A, R  A |
| ENSMUST00000177840.9 | O35566 |  |  |  |  |  |
| ENSMUST00000058746.7 | O35566 |  |  |  |  |  |
| **CD63** |  |  |  |  |  |  |
| **Transcript** | **UniProt Match** | **Gene** | **Expression** | **Rank score** | **Expression score** | **Sources** |
| ENSMUST00000219317.2 | P41731, Q549D0 | ENSMUSG00000025351 | Secondary oocyte | 2.08e4 | 50.00 | R |
| ENSMUST00000026407.9 | P41731, Q549D0 |  |  |  |  |  |
| ENSMUST00000105229.9 | P41731, Q549D0 |  |  |  |  |  |

| **CD82** |  |  |  |  |  |  |
| --- | --- | --- | --- | --- | --- | --- |
| **Transcript** | **UniProt Match** | **Gene** | **Expression** | **Rank score** | **Expression score** | **Sources** |
| ENSMUST00000028644.11 | P40237, Q3UII2 | ENSMUSG00000027215 | Primary oocyte  Secondary oocyte  Cumulus cells | 8.23e3  9.02e3  8.24e3 | 80.26  78.36  80.22 | A, R  A, R  A |
| ENSMUST00000099696.8 | P40237, Q3UII2 |  |  |  |  |  |
| ENSMUST00000111256.8 | A2AIJ3 |  |  |  |  |  |
| ENSMUST00000111257.8 | P40237, Q3UII2 |  |  |  |  |  |
| ENSMUST00000116457.9 | P40237, Q3UII2 |  |  |  |  |  |
| ENSMUST00000123565.8 | A2AIJ1 |  |  |  |  |  |
| ENSMUST00000124804.3 | A2AIJ4 |  |  |  |  |  |
| ENSMUST00000145553.8 | A2AIJ2 |  |  |  |  |  |
| ENSMUST00000150508.8 | A2AIJ2 |  |  |  |  |  |

**Transcript IDs and Genes** are listed in Ensembl ID format (<https://www.ensembl.org/>). **UniProt Match** refers to the UniProt identifier of protein (<https://www.uniprot.org/>) that corresponds to the Ensembl transcript. Only transcripts encoding the protein were included. **Expression** data were retrieved from the Bgee database (release 14.2) (<http://bgee.org/>). **Rank scores** of expression calls are normalized across genes, conditions, and species. A low score means that the gene is highly expressed in the condition. Max rank score in all species: 4.10e4. Min rank score varies across species. **Expression scores** of expression calls use the minimum and maximum Rank of the species to normalize the expression to a value between 0 and 100. A low score means that the gene is lowly expressed in the condition. Sources of data: A-Affymetrix, R-RNA-Seq. Not listed cell types and tissues have not been tested.

**Supplementary Table 4 Summarized data of in silico analysis of expression of CD9, CD81, CD151, CD63, and CD82 tetraspanin trascripts in cells of ovary and oocytes**

|  |  | **Species** | | | |
| --- | --- | --- | --- | --- | --- |
| **Tetraspanin** | **Expression/localization of tetraspanin** | ***Bos taurus*** | ***Homo sapiens*** | ***Sus scrofa*** | ***Mus musculus*** |
| **CD9** | Ovary | NT | + | NT | NT |
|  | Germinal epithelium of ovary | NT | + | NT | NT |
|  | Theca cells | **+** | NT | NT | NT |
|  | Granulosa cells | **+** | NT | + | NT |
|  | Cumulus cells | + | NT | NT | + |
|  | Oocyte | NT | + | - | + |
| **CD81** | Ovary | NT | + | NT | NT |
|  | Germinal epithelium of ovary | NT | + | NT | NT |
|  | Theca cells | + | NT | NT | NT |
|  | Granulosa cells | + | NT | + | NT |
|  | Cumulus cells | + | NT | NT | + |
|  | Oocyte | NT | + | - | + |
| **CD151** | Ovary | NT | + | NT | NT |
|  | Germinal epithelium of ovary | NT | + | NT | NT |
|  | Theca cells | + | NT | NT | NT |
|  | Granulosa cells | + | NT | + | NT |
|  | Cumulus cells | + | NT | NT | + |
|  | Oocyte | NT | - | + | + |
| **CD63** | Ovary | NT | + | NT | NT |
|  | Germinal epithelium of ovary | NT | + | NT | NT |
|  | Theca cells | + | NT | NT | NT |
|  | Granulosa cells | + | NT | + | NT |
|  | Cumulus cells | + | NT | NT | NT |
|  | Oocyte | NT | + | + | + |
| **CD82** | Ovary | NT | + | NT | NT |
|  | Germinal epithelium of ovary | NT | + | NT | NT |
|  | Theca cells | + | NT | NT | NT |
|  | Granulosa cells | + | NT | + | NT |
|  | Cumulus cells | + | NT | NT | + |
|  | Oocyte | NT | **+** | **-** | **+** |

Summarized data of in silico analysis of expression/localization of CD9, CD81, CD151, CD63, and CD82 tetraspanin transcripts in cells of ovary and oocytes using databases: Ensembl ID (<https://www.ensembl.org/>), **UniProt Match** (<https://www.uniprot.org/>) and Bgee database (release 14.2) (<http://bgee.org/>). Not listed cell types and tissues have not been tested.

**+** refers to available expression data,  **-** refers to absent expression, and NT refers that cells (tissue) were not tested.
